# Invasive guests: the role of non-oral bacteria in oral dysbiosis and systemic health

**DOI:** 10.1080/20002297.2026.2745592

**Published:** 2026-10-18

**Authors:** Marzie Mahdizade Ari, Ali Al-Ahmad, Fabian Cieplik, Konstantin Johannes Scholz

**Affiliations:** a Department of Microbiology, School of Medicine, Iran University of Medical Sciences, Tehran, Iran; b Department of Operative Dentistry and Periodontology, Center for Dental Medicine, Faculty of Medicine and Medical Center, University of Freiburg, Freiburg im Breisgau, Germany

**Keywords:** Non-oral bacteria, oral microbiome, oral dysbiosis, microbial colonization, opportunistic pathogens, biofilm formation, antibiotic resistance

## Abstract

**Background:**

The human oral cavity harbors a diverse microbiota essential for maintaining oral and systemic health. While most resident microorganisms are commensals adapted to specific oral niches, increasing evidence highlights the presence of non-oral bacteria, including *Helicobacter pylori, Pseudomonas aeruginosa, Klebsiella pneumoniae, Enterobacteriaceae, Staphylococcus aureus, Acinetobacter baumannii, Enterococcus faecalis* and *Clostridioides difficile*. Traditionally associated with other body sites, these bacteria may enter the oral cavity and, under favorable ecological conditions, persist through mechanisms such as biofilm formation, stress tolerance, entry into a viable but non-cultivable (VBNC) state, coaggregation with the oral microbiota, and evasion of host immune responses. Their colonization has been reported particularly in dysbiotic oral environments, including periodontitis and immunocompromised conditions, where overgrowth may also facilitate their recovery by conventional culture methods.

**Objective:**

This review aims to summarize the current evidence on the presence, persistence mechanisms, clinical significance, and detection challenges of non-oral bacteria in the oral cavity, and to highlight the need for integrated diagnostic approaches.

**Design:**

A comprehensive literature review was conducted focusing on studies reporting the isolation, detection, and clinical relevance of non-oral opportunistic bacteria in the oral cavity, with emphasis on culture-based and molecular diagnostic methods.

**Results:**

Clinically, non-oral bacteria presence has been associated with dental caries, periodontal disease, endodontic infections, systemic dissemination, and their potential role as reservoirs of antibiotic resistance genes. Although several non-oral opportunistic bacteria can be readily isolated by culture when present at sufficient abundance, particularly in immunocompromised patients, detection may remain challenging in healthy individuals or low-biomass samples because of their transient or low-abundance occurrence, increasing the risk of misinterpretation.

**Conclusions:**

Recognizing the oral cavity as a dynamic niche for non-oral bacteria underscores the need for integrated culture- and molecular-based diagnostic approaches, improved infection control strategies, and further research into their pathogenic potential.

## Key messages

- The oral cavity functions not only as a habitat for resident microbiota but also as a dynamic ecological interface where non‑oral microorganisms can transiently or persistently colonize and integrate into biofilms. They may contribute to oral dysbiosis, with the balance between transient and persistent presence remaining organism‑specific.
- Through adaptive traits such as biofilm formation, VBNC transition, niche exploitation, and horizontal gene transfer, several non‑oral bacteria appear capable of persisting in the oral environment and acting as reservoirs of antimicrobial resistance, though the strength of this evidence varies markedly by organism.
- The presence of non‑oral bacteria in oral samples challenges conventional diagnostic paradigms, necessitating microbiome‑aware interpretation and standardized molecular approaches to distinguish true colonization from contamination.

## Introduction

The human oral cavity hosts a complex and highly specialized microbial ecosystem known as the oral microbiome, which plays a vital role in maintaining oral and systemic health through balanced microbial interactions [1]. Traditionally, this microbiome was considered to consist primarily of microorganisms adapted to distinct oral niches, such as the tongue, gingiva, and dental plaque. However, increasing evidence has revealed the presence of non-oral microorganisms, species typically associated with other body sites or environmental sources, raising important questions about microbial transmission, adaptation, and disease [2]. Disruption of the oral microbiome by factors, including poor oral hygiene, unhealthy diet, tobacco or alcohol use, systemic diseases, and antimicrobial therapies, may facilitate colonization by opportunistic non-oral pathogens, such as Gram-negative enteric rods, *Enterococci* and *Staphylococci* [3]. These organisms have been detected with oral diseases, including periodontitis, apical periodontitis, and peri-implantitis [4], while the oral cavity may also serve as a reservoir for their dissemination to distant organs, contributing to conditions such as pneumonia, endocarditis, and gastrointestinal infections [5].

Despite increasing evidence, the mechanisms underlying non-oral bacteria persistence and ecological adaptation remain incompletely understood. Given the scarcity of comprehensive studies on this topic, the detection of non-oral bacteria in the oral cavity, such as Gram-negative enteric rods, *Helicobacter pylori*, *P. aeruginosa*, *S. aureus*, and others, is of significant concern [6]. While our previous review highlighted the viable but non-culturable (VBNC) state as one of the bacterial survival strategies within the oral microbiota [7], the present review broadens this question to ask whether non‑oral and opportunistic bacteria can establish transient or persistent colonization within the oral ecosystem, and to investigate their clinical significance, diagnostic challenges, and their potential role as reservoirs of antimicrobial resistance.

### Search strategy and scope

Relevant literature was retrieved through PubMed and Google Scholar using combinations of the microorganisms discussed (e.g. *Helicobacter pylori, Pseudomonas aeruginosa, Klebsiella pneumoniae, Enterobacteriaceae, Staphylococcus aureus, Acinetobacter baumannii, Enterococcus faecalis, and Clostridioides difficile*) with terms including oral cavity, oral microbiome, non-oral bacteria, opportunistic pathogens, biofilm, viable but non-cultivable and antibiotic resistance. Searches were unrestricted by publication date until 31st July, 2026. Only full-text articles in the English language were included, and additional relevant studies were identified by screening reference lists. Studies were selected based on their relevance to the detection, persistence, colonization, ecological adaptation, and clinical significance of non-oral bacteria in the oral cavity.

### Natural colonizers of the oral cavity

The human oral cavity harbors a complex microbial ecosystem comprising more than 700 bacterial species, together with fungi, viruses, protozoa, and archaea [8]. The composition and balance of this microbiota are influenced by oral hygiene, diet, systemic health and environmental factors, such as diabetes mellitus, antibiotic use, and smoking [9]. Disruption of this balance (microbial dysbiosis) has been associated with dental caries and periodontal diseases, but also oral cancer [10] and several systemic diseases through inflammation, immune modulation, and altered microbial interactions [11]. Pathogenicity may also vary within individual taxa; for example, certain *Fusobacterium nucleatum* subspecies have been reported to be enriched in oral squamous cell carcinoma and exhibit enhanced virulence [12].

Dominant bacterial genera include *Streptococcus, Actinomyces, Fusobacterium, Porphyromonas,* and *Veillonella*. Although these organisms generally function as commensals, their roles are highly dependent on ecological conditions such as nutrient availability, oxygen tension, host immunity, and biofilm architecture [13]. Under dysbiotic conditions, acidogenic species such as *Streptococcus mutans* are associated with dental caries, whereas proteolytic anaerobes, including *Porphyromonas gingivalis* are associated with periodontal diseases [14]. In addition to bacteria, fungi (e.g. *Candida albicans*) [15], protozoa (e.g. *Entamoeba gingivalis* and *Trichomonas tenax*) [16], and viruses [17], also contribute to oral health and disease.

### Factors that determine bacterial preference for the oral cavity

The oral cavity represents the second most diverse microbial habitat in the human body after the gut, with core genera dominating across individuals and exhibiting niche‑specific distributions in saliva, supra‑ or subgingival dental plaque, the tongue, and infected root canal systems [18] according to local oxygen levels, pH, nutrient availability, and mechanical stress [19]. Whole‑genome sequencing (WGS) of seven distinct oral microhabitats from healthy subjects has confirmed that each niche harbors a distinct microbial community, with alpha diversity differing significantly among oral sites but not between healthy individuals, supporting the existence of a conserved healthy core microbiome despite niche‑specific ecological variation [20]. Shotgun metagenomic analyses have further demonstrated that oral community structure is primarily determined by habitat rather than host characteristics, with beta‑diversity analyses confirming strong site‑specific microbial organization [21]. Anaerobes such as *Fusobacterium* spp. typically inhabit subgingival plaque, while facultative species such as *Streptococcus* spp. prevail supragingivally [22]. Microbial adhesion mechanisms, including adhesins, pili, and biofilm formation, enable colonization and persistence under environmental and immune pressures [23]. Collectively, host and environmental factors shape microbial diversity and biofilm stability [15]. Frequent sugar consumption favors acidogenic and aciduric species such as *S. mutans*, promoting low‑pH environments and dental caries [24]. Disturbances in these factors, such as poor oral hygiene or systemic diseases, lead to dysbiosis, contributing to caries, gingivitis, periodontitis, and possibly systemic conditions, including cardiovascular disease and diabetes [25]. Interspecies interactions, including competition, bacteriocin production, and metabolic cooperation, further stabilize oral biofilms and help maintain microbial balance [13].

### Unexpected guests: non-oral bacteria in the oral cavity

The oral cavity, in addition to its resident microbiota, can also harbor non-oral bacteria originating from the gastrointestinal tract, skin, or environment, and their migration across body sites has increasingly been linked to chronic systemic diseases [26]. In microbial ecology, colonization is classically defined as the persistence of microorganisms on a body surface without causing disease, in contrast to infection, which involves tissue invasion and a host response [27]. A central question is whether the detection of such organisms in the oral cavity reflects incidental contamination, transient passage, or genuine long-term colonization. At present, robust evidence meeting these criteria remains limited: although repeated molecular detection across independent sampling time points, genetic divergence of oral strains, and evidence of host interaction may suggest persistence rather than one-off contamination [28], these findings alone do not prove stable colonization. Given the scarcity of viable isolates and the predominantly correlative nature of current evidence, often derived from molecular, culture, or animal studies, any claim of persistence should be viewed with due caution.

In practice, however, the operational definition of colonization depends strongly on both temporal and spatial sampling. Repeated sampling substantially alters estimated colonization rates, as demonstrated for *C. difficile* [29], while organisms inconsistently detected in pooled saliva may exhibit stable persistence when individual oral niches, such as periodontal pockets, are sampled longitudinally [30].

Colonization evidence varies by organism. *P. aeruginosa* and *S. aureus* are readily cultivable from saliva and subgingival plaque samples and exhibit robust biofilm-forming capacity [31], whereas *H. pylori* has been detected in subgingival plaque samples predominantly by molecular methods without the recovery of viable oral isolates [32]. *E. faecalis* is a well-established endodontic colonizer [33], whereas *C. difficile* has so far been identified only molecularly within subgingival plaque samples [34].

Successful colonization generally depends on both microbial traits and host environmental conditions. Colonization is most likely to occur under conditions of host immune impairment or oral dysbiosis, where inflammation, altered host defenses, and changes in nutrient availability facilitate the integration of non-oral bacteria into established biofilms [35,36]. Persistent detection of species such as *P. aeruginosa*, *A. baumannii*, and *E. faecalis* has been reported in susceptible individuals, often in association with disease progression [37]. Collectively, these findings challenge the traditional concept of strict tissue-specific microbial residency, suggesting that the oral cavity should be viewed as a dynamic ecosystem shaped by both resident and transient microorganisms.

#### Helicobacter pylori

*Helicobacter pylori* is a microaerophilic bacterium strongly associated with peptic ulcers, gastric cancer, and MALT lymphoma [38], with its eradication increasingly complicated by the global rise of antibiotic resistance [39]. Transmission occurs via oral‑oral, fecal‑oral, and gastro‑oral routes [40]. *H. pylori* is frequently detected by molecular methods in dental plaque, saliva, tongue coating, carious lesions, and occasionally on the tongue dorsum and buccal epithelium [41–43]. Genotypes identified from oral samples often resemble those found in gastric biopsies [44,45]. However, no viable oral isolates have yet been recovered, and the evidence for active oral colonization remains indirect. Thus, the oral cavity has been proposed as a potential transient source of gastric re-exposure, although this hypothesis remains speculative and requires direct experimental validation [46]. A recent PCR-based study detected *H. pylori* DNA in 20.6% of saliva samples, including healthy controls, but emphasized that PCR cannot distinguish viable colonization from transient contamination, with detection rates substantially higher in dental plaque than in saliva [47], consistent with current evidence supporting transient persistence rather than stable colonization. Nevertheless, the available evidence remains conflicting. While some meta-analyses have suggested that *H. pylori* may persist within periodontal pockets by exploiting localized anaerobic microenvironments [48], others argue that its strict microaerophilic requirements are more consistent with transient oral persistence than stable colonization [49]. The persistent inability to recover viable oral isolates may partly reflect the bacterium's capacity to enter a VBNC state under environmental stress [7,41]. Under *in vitro* conditions, *H. pylori* survived in human saliva for at least 48 h when co-cultured with *S. mutans, A. naeslundii* and *C. dubliniensis*, indicating that polymicrobial communities may facilitate bacterial survival during transmission [50].

The oral cavity presents several hostile conditions for *H. pylori*, including low CO₂ concentrations, fluctuating oxygen tension and temperature, continuous salivary flow [51], and *in vitro* antagonistic activity of oral *streptococci* has been demonstrated [52]. To overcome these challenges, the bacterium relies on motility, adhesins (BabA and SabA), urease activity, and virulence factors such as CagA and VacA, which promote adhesion and interfere with host cell signaling [53,54]. Biofilms within carious lesions and periodontal pockets provide protected microenvironments with favorable nutrient and gas gradients [51], while interactions with *P. gingivalis, F. nucleatum,* and *C. albicans* enhance persistence [55]. In contrast, *S. mitis* and *Prevotella intermedia* inhibit bacterial growth and promote transformation into the coccoid form [56]. Remarkably, *H. pylori* can replicate within *C. albicans* and undergo vertical transmission [57].

*H. pylori* employs multiple mechanisms to evade host defenses. Urease-mediated pH regulation, lipopolysaccharide (LPS) modification to reduce TLR4 recognition [55], and suppression of macrophage phagocytosis and T-cell activation [56] facilitate immune evasion. Intracellular localization within periodontal ligament fibroblasts induces G2 cell-cycle arrest and impaired proliferation [58]. Host cell receptors may also contribute to persistence. In gastric epithelial cells, increased CEACAM expression enhances bacterial adhesion and CagA translocation, and because CEACAM expression is elevated in periodontitis and oral squamous cell carcinoma independently of *H. pylori*, it is plausible that similar mechanisms could facilitate bacterial persistence in oral tissues [59]. However, the relevance of these mechanisms to oral tissues remains speculative and needs experimental validation.

#### Pseudomonas aeruginosa

*P. aeruginosa* is an opportunistic pathogen with a remarkable ability to form biofilms, which enhances persistence and antimicrobial tolerance [60]. These biofilms, supported by extracellular polymeric substances (EPS), limit antibiotic penetration and immune clearance, while efflux pumps and horizontal gene transfer further strengthen possible drug resistance [61]. Although *P. aeruginosa* is primarily known for its environmental persistence and healthcare‑associated infections, it has also been detected infrequently in the oral cavity, including subgingival plaque, saliva, dorsum of the tongue and buccal mucosa [31,62]. While the association between periodontal disease and pneumonia remains debated, oral colonization by *P. aeruginosa* is common in hospitalized and medically compromised individuals [63], and evidence suggests migration from the oral cavity to the lower respiratory tract [64]. Consequently, poor oral health is considered a risk factor for nosocomial pneumonia regardless of mechanical ventilation [65]. Contamination is often linked to dental unit waterlines (DUWLs), which represent major reservoirs of opportunistic pathogens, including *P. aeruginosa, Legionella* spp., and nontuberculous *mycobacteria* [66], with narrow tubing, temperature, low flow, and stagnation facilitating biofilm formation, and municipal water, bottled water, or patient backflow acting as contamination sources [67]. Within DUWL biofilms, *P. aeruginosa* persists despite disinfectants and intermittently releases planktonic cells into treatment water, which can be aerosolized during dental procedures, posing risks, particularly to immunocompromised patients and staff [68].

In addition to its role in the oral cavity, *P. aeruginosa* can contribute to salivary gland stones (sialoliths) formation [69]. The bacterial communities within sialoliths, mainly *Streptococcus* spp., sometimes include *P. aeruginosa*, and biofilm structures may promote inflammation, tissue damage, and glandular calcification [70], with bacteria‑positive stones often associated with recurrent or severe sialadenitis [71]. The carbapenem-resistant *P. aeruginosa* is associated with increased mortality in lower respiratory infections globally [72]. Notably, multidrug‑resistant (MDR) *P. aeruginosa* is increasingly reported in oral infections, especially among immunocompromised patients [61], and a biofilm‑producing isolate from dental caries, *P. aeruginosa* DC‑17, showed strong EPS‑mediated biofilm stability and high resistance to several antibiotics, though it remains sensitive to amoxicillin [73]. Environmental conditions such as elevated temperature, occasionally alkaline pH, and high sugar levels further enhanced its biofilm formation under oral conditions [74]. Collectively, these findings indicate that *P. aeruginosa*, although not a typical oral resident, can establish resilient biofilms and act as a reservoir for opportunistic infections when the oral ecological balance is disrupted.

#### Klebsiella pneumoniae

*Klebsiella pneumoniae*, a highly virulent member of the ESKAPE pathogens (*E. faecium*, *S. aureus*, *K. pneumoniae*, *A. baumannii*, *P. aeruginosa* and *Enterobacter* species) and the family *Enterobacteriaceae*, is well known for its multidrug‑resistance and as a major cause of nosocomial infections [75]. The oral cavity can serve as a reservoir of resistance‑ or hypervirulence‑associated genes of *K. pneumoniae*, as observed in samples from hospitalized patients [76]. *K. pneumoniae* can typically be found in the environment (soil, water, and animals), in endogenous human niches (gastrointestinal tract and skin), and contaminated healthcare settings [77]. Although primarily associated with gastrointestinal, hospital-acquired respiratory and urinary infections [78], *K. pneumoniae* has also been detected in the oral cavity, including periodontal pockets, saliva, and dorsum of the tongue [79], typically at low abundance and in a limited subset of individuals. Its presence and potential association with periodontitis have been reported [80], but current evidence remains insufficient to establish *K. pneumoniae* as a major periodontal pathogen [81]. Animal studies have demonstrated that oral *Klebsiella* spp. can translocate to the gut, leading to inflammation and colitis [82], and although not commonly found in the mouth, when present, it tends to establish stable colonization [83]. This is clinically significant, as even a small number of carriers could facilitate transmission in healthcare environments [84].

*K. pneumoniae* demonstrates remarkable resilience under adverse conditions, tolerating limited nutrient supply, pH variations, desiccation, and other stresses through biofilm formation, protection by a thick polysaccharide capsule, and stress‑response mechanisms such as signal transduction pathways [85]. Biofilms develop not only on host tissues but also on abiotic surfaces such as medical devices and hospital environments [86], including dry surface biofilms (DSBs) that are difficult to eradicate [87], and are a possible source of airborne contamination without appearing in the oral cavity directly. Complementing these survival strategies, a recent microcosm study showed that *Klebsiella* spp. can survive antiseptic stress, with *K. oxytoca* from microcosm biofilms inoculated from human saliva growing on chlorhexidine (CHX)‑ and cetylpyridinium‑containing media, indicating intrinsic or acquired tolerance [88]. These isolates also exhibit multidrug resistance (aminopenicillins, piperacillin and fosfomycin) via mechanisms such as AmpC *β*-lactamase and fosA, highlighting their potential for long-term persistence in oral and nasal niches. *K. pneumoniae* can endure prolonged starvation, outlasting many other bacteria for over 100 days and rapidly regrowing when nutrients become available [86]. Genomic analyses corroborate this adaptability, showing that oral and nasal isolates are closely related to clinical and environmental strains and harbor mobile genetic elements, virulence factors, and resistance determinants [85]. Although oral colonization appears relatively uncommon, persistence is supported by factors such as adhesion, immune evasion, microbial competition, and environmental conditions (e.g. humidity) [89]. Notably, while suppressed by a healthy salivary microbiota, *K. pneumoniae* can expand under dysbiotic and nutrient-limited conditions [22], linking its environmental robustness with its capacity to maintain and spread resistance traits.

### Other members of Enterobacteriaceae (*Escherichia coli*, *Serratia* species)

*Enterobacteriaceae,* as Gram-negative enteric rods (GNRs), may be introduced into the oral cavity via ingestion of contaminated food or water, as well as through poor public and personal hygiene practices, most notably hand-to-mouth transmission following contact with contaminated surfaces, rather than as a consequence of inadequate oral hygiene alone [6]. Their pathogenicity is mediated by a combination of virulence factors, including toxin production, immune evasion strategies, and tissue-degrading enzymes that facilitate survival and persistence within the oral environment [6]. Although not considered core members of the oral microbiota, *E. coli*, *Klebsiella* spp., *Enterobacter* spp., and *Serratia* spp. can act as opportunistic pathogens under conditions of ecological disruption [90]. Notably, they can survive exposure to common oral antiseptics and frequently exhibit resistance to multiple antibiotics, making the oral cavity a potential reservoir for resistant *Enterobacteriaceae* under dysbiotic conditions or repeated antiseptic exposure [88].

*E. coli* is a common gut commensal that generally exerts beneficial effects, including vitamin B and K synthesis and colonization resistance against pathogens such as *Salmonella* spp. through the suppression of their growth and virulence [91,92]. In contrast, pathogenic *E. coli* lineages are responsible for a broad spectrum of infections, including diarrhea, urinary tract infections, sepsis, pneumonia, and bacteremia [6]. In the oral cavity, *E. coli* has been detected in endodontic infections, abscesses, buccal mucosa, supragingival plaque, and the tongue dorsum [93], with its presence often attributed to transient colonization or fecal–oral transmission, particularly among children [94], *E. coli* and *K. pneumoniae* have been identified as the predominant Gram‑negative species [88], in contrast to *Streptococcus pneumoniae* as the most frequent Gram‑positive isolate in dental specimens [95]. *E. coli* has also been detected in oral lichen planus (OLP), a chronic T cell‑mediated inflammatory disorder associated with altered microbial communities [96]. Strains isolated from OLP lesions were genetically distinct from fecal isolates, possessing aggregation and DNA‑transfer genes absent in laboratory strains, and unlike classical pathogenic *E. coli*, these OLP‑associated isolates exhibited smaller genomes with reduced virulence repertoires [97]. Their presence in both healthy and diseased oral tissues supports a pathobiont role, as commensals that can contribute to disease under immune dysregulation or epithelial barrier disruption [93].

*Serratia liquefaciens* and *Enterobacter cloacae* have also been detected in saliva, with prevalence rates of 31.8 and 18.1% in a cohort of students from Northeast Brazil [98], and their occurrence is influenced by systemic factors such as aging, poor general hygiene, and immunosuppression, with higher prevalence in HIV‑positive individuals [99]. Oral inflammation promotes their growth, while their pro‑inflammatory factors disrupt epithelial barriers and may contribute to systemic conditions, including respiratory, metabolic, renal, cardiovascular, neurological disorders, obesity and cancer [90]. Colonization is further affected by age [100], orthodontic appliances [101], and periodontal pocket depth [102].

Oral bacteria play a significant role in kidney complications, particularly in chronic kidney disease (CKD). In patients with periodontitis, *P. gingivalis, A. actinomycetemcomitans* and *F. nucleatum* have been discussed to induce kidney damage and contribute to CKD progression via transient bacteremia, circulating microbial toxins, and systemic inflammation, which, together with uremic toxin accumulation, suppress immunity and increase susceptibility to infections, including peritonitis and exit‑site infections in CKD patients undergoing peritoneal dialysis (CKD‑PD) [103]. Similarly, oral *Enterobacteriaceae*, including *R. ornithinolytica, K. pneumoniae, K. oxytoca, E. coli* and *E. asburiae*, further elevate this risk. The altered oral environment in CKD‑PD patients favors colonization, with 31.6% MDR strains detected exclusively in this group, contributing to 8% of peritonitis cases and 16% of exit‑site infections [104].

#### Staphylococcus aureus

*Staphylococcus aureus* is a well‑known pathogen frequently associated with hospital‑acquired and opportunistic infections, but it also contributes to oral health complications, particularly in immunocompromised individuals. Oral colonization has been confirmed at various sites, including gums, tongue, saliva, mucosa, and removable dental appliances, and is strongly linked to denture stomatitis [105]. Colonization may originate from the oral cavity itself or via transmission from the anterior nares through the oropharynx [106]. In dental settings, the risk of cross‑transmission between patients and staff is elevated, mostly through inhalation of bacteria‑containing droplets generated by dental instruments [107]. Oral carriage rates vary widely, ranging from 24 to 84% in healthy adults [108] and approximately 48% in denture wearers [109], with older adults being particularly vulnerable due to age‑related immune decline, chronic conditions, medications, or nutritional deficiencies [110]. Additionally, broad‑spectrum antibiotic use can disrupt the oral microbiota, favoring *S. aureus* colonization [111].

Recent reports indicate increasing carriage rates of both methicillin‑susceptible (MSSA) and methicillin‑resistant *S. aureus* (MRSA) in the oral cavity, with MSSA remaining more prevalent overall among patients, healthcare workers, and community samples [112]. Carriage often occurs simultaneously in nasal and oral cavities, although resistance profiles may differ between sites, with multidrug resistance more frequently observed in oral isolates [113]. Prevalence varies with population characteristics, geography, sampling methods, and healthcare exposure [114]. Notably, MRSA colonization has also been detected in individuals without any clinical contact, and 19% of MRSA‑positive patients show oral but not nasal colonization, suggesting that the oral cavity may act as an independent reservoir for antimicrobial‑resistant *S. aureus*. This finding raises concerns about persistent colonization despite nasal decolonization strategies [115,116]. Although MRSA is rarely reported as a direct cause of oral disease, its resistance to common antibiotics such as amoxicillin challenges the effectiveness of current prophylactic dental protocols, which are now limited to patients with specific cardiac conditions [112,113].

#### Acinetobacter baumannii

*Acinetobacter baumannii*, a Gram‑negative opportunistic pathogen, can persist in the oral cavity, including subgingival plaque, biofilms, and oral mucosa, where it integrates into complex multispecies biofilms and often coexists with key periodontitis‑associated bacteria (*P. gingivalis, T. forsythia, T. denticola* and *A. actinomycetemcomitans*), suggesting a potential role in periodontal disease progression [81]. Early colonizers of dental hard tissue surfaces, such as *S. sanguinis*, produce bactericidal compounds that can inhibit its growth, while Gram‑negative anaerobes support its establishment [117]. The oral cavity may serve as a reservoir for dissemination into the lower respiratory tract, contributing to pneumonia and COPD. *A. baumannii* is a leading cause of ventilator-associated pneumonia (VAP), particularly in ICUs, and oral hygiene interventions, including cleansing with 0.2% CHX, have been shown to reduce VAP risk [118]. Several virulence mechanisms have been proposed to promote *A. baumannii* persistence: Type IV pili and biofilm‑associated protein (Bap) facilitate attachment and biofilm maturation [119], outer membrane protein A (OmpA) induces host cell apoptosis in cell culture models, the polysaccharide capsule and acinetobactin‑mediated iron acquisition have been characterized in clinical isolates [120], and the type VI secretion system (T6SS) deliver toxic effectors to competing microbes under laboratory conditions. However, direct evidence for the *in vivo* contribution of these mechanisms to oral colonization in humans remains limited. *A. baumannii* can inhibit *C. albicans* biofilms while inducing fungal apoptosis, highlighting interkingdom interactions, and co‑colonization with *P. aeruginosa* has been linked to aggressive periodontitis, with both organisms communicating via acyl‑homoserine lactone (AHL) quorum sensing [121].

#### Enterococcus faecalis

Enterococci, particularly *E. faecalis*, are generally considered transient inhabitants rather than stable members of the oral microbiome. Their prevalence in healthy individuals is low (1–17%), but they are frequently detected in endodontic infections and mucosal surfaces in immunocompromised or hospitalized patients, and particularly in cases of persistent or repeated reinfection [122], periodontal diseases, and other oral pathologies [123]. Entry into the oral cavity occurs via contaminated or fermented foods such as cheese [124], occupational exposure to meat, hospital contact, and dental procedures, while poor oral hygiene and necrotic or treated root canals provide ecological niches for colonization [125]. Periodontal pockets, with anaerobic and nutrient‑rich conditions, are particularly favorable for transient establishment [33,126]. Once introduced, *E. faecalis* adheres to oral surfaces via surface and collagen‑binding proteins and forms biofilms on both biotic (dentin and periodontal tissues) and abiotic surfaces, enhancing resistance to mechanical clearance by saliva or hygiene measures [127,128]. In healthy individuals, colonization is usually short‑lived and influenced by age, sex, diet, and oral health, with children harboring fewer enterococci and females showing a slightly higher prevalence [123]. Under pathological conditions, particularly in endodontic or periodontal infections, *E. faecalis* can persist and act as a reservoir for secondary or recurrent infections, especially in treated root canals [33]. Nevertheless, a clinical study of healthy volunteers who consumed cheese containing enterococci showed that *E. faecalis* from cheese was able to integrate into the supragingival oral biofilm *in vivo* [124], suggesting that endodontic infections could originate from such fermented foods.

*E. faecalis* survives in the oral cavity due to its remarkable tolerance to environmental stresses, including wide temperature (10–45 °C), high salinity, variable pH, ethanol, and hydrogen peroxide [129]. Moreover, in endodontics, its resistance to calcium hydroxide is particularly important, attributed to its ability to tolerate highly alkaline environments [130]. Sub‑lethal stress exposure induces cross‑protection, enhancing resistance to oxidative stress, hyperosmolarity and intracanal antimicrobials [125]. Structural adaptations, including a thickened peptidoglycan layer, teichoic acid‑containing wall, and proton pumps regulating the intracellular pH, further support resistance to alkaline medicaments and osmotic stress. Biofilm formation enhances adhesion and provides robust protection against antibiotics and disinfectants [128]. Additionally, *E. faecalis* expresses virulence factors such as gelatinase, hemolysin, cytolysins, and extracellular superoxide, contributing to tissue damage, inflammation, and potential systemic dissemination via periodontal sites. This combination of mechanisms underlies *E. faecalis’s* persistence and its capacity for recurrent oral and systemic infection despite host defenses and treatment [126,127].

#### Clostridium difficile

*Clostridioides difficile* infection (CDI) is a major global health concern with significant clinical and economic impact. Disruption of the gut microbiota enables *C. difficile* colonization, toxin production, and disease ranging from mild diarrhea to severe colitis, with changes in epidemiology driven by overuse and misuse of antibiotics, antimicrobial resistance, and the COVID‑19 pandemic having increased disease burden [131]. The oral cavity represents a major gateway for microbial entry into the lower gastrointestinal tract, including *C. difficile*. [132]. As a member of the *Peptostreptococcaceae* family, *C. difficile* has been shown to colonize anaerobic subgingival biofilms and is highly abundant in patients with periodontal disease; however, it has not been isolated from the oral cavity using culture‑based methods, with detection so far achieved only through molecular techniques [34]. It has also been identified among the predominant taxa in root canal infections associated with periapical lesions refractory to endodontic therapy [133]. Moreover, an atypical case of septicemia with a suspected oral origin has been reported, without any association between fecal and blood isolates or underlying gastrointestinal disease, in a patient who had undergone traumatic tooth extraction due to caries shortly before symptom onset, though the periodontal status was not assessed [134]. Molecular studies further support the presence of *C. difficile* in periodontal sites, with positive testing in over 20% of subgingival biofilm samples from patients with advanced periodontitis [135,136]. From a clinical perspective, the persistence of *C. difficile* vegetative cells or spores within the subgingival biofilm may reduce the effectiveness of antimicrobial treatment for CDI, positioning subgingival biofilms as potential reservoirs for oral–fecal reinfection via saliva [34]. Dentists contribute approximately 6–10% of outpatient antibiotic prescriptions and frequently prescribe clindamycin, an agent associated with an increased risk of CDI [137]. Although the overall likelihood of developing symptomatic CDI remains low, repeated or prolonged clindamycin exposure may facilitate asymptomatic oral carriage in susceptible populations, including individuals with periodontitis. [138]. Consistently, a correlation between oral and fecal carriage of *C. difficile* has been observed in saliva, but not in subgingival biofilm samples, suggesting that saliva may act as a transient carrier facilitating the transfer of *C. difficile* cells or spores to the gut microbiota.

## Discussion

### How do non-oral bacteria get and survive in the oral cavity?

After initial colonization, aerobic and facultative anaerobic species such as *Streptococcus* spp., *Actinomyces* spp., *Veillonella* spp., and *Neisseria* spp. dominate within biofilms on oral surfaces (tongue, teeth), while obligate anaerobes such as *Prevotella* spp. and *Fusobacterium* spp. emerge following tooth eruption as anaerobic niches develop [139,140]. Despite a stable resident community, the oral cavity remains exposed to exogenous microorganisms from food, water, air, and person‑to‑person contact [141]. Non‑native bacteria may enter the oral cavity through several distinct pathways (Figure 1).

**Figure 1.**
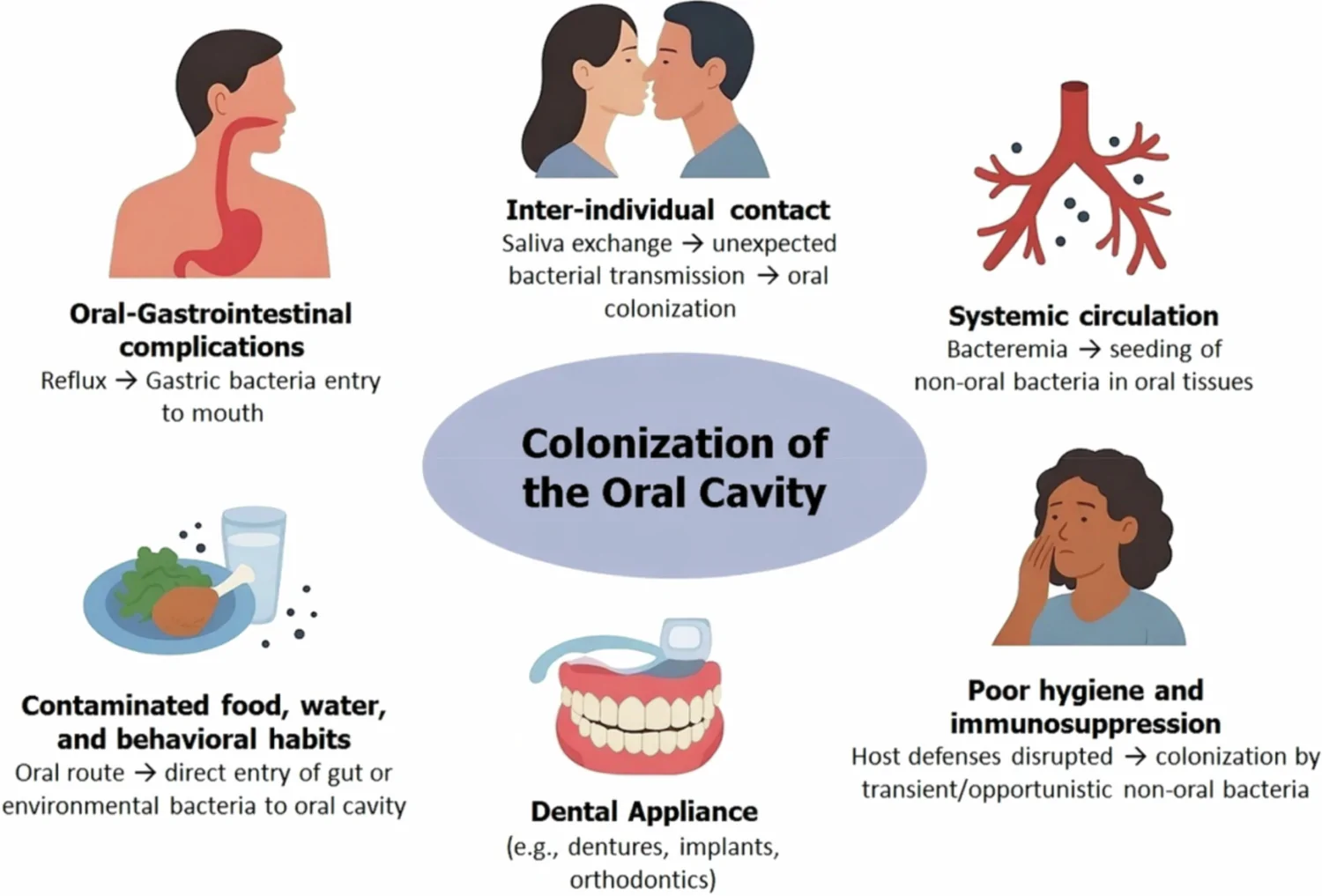
Six routes by which non-native bacteria enter the oral cavity and colonize it: inter-individual contact, in which saliva exchange enables bacterial transmission and oral colonization; systemic circulation, in which bacteremia seeds non-oral bacteria into oral tissues; poor hygiene and immunosuppression, in which disrupted host defenses permit colonization by transient opportunistic non-oral bacteria; dental appliances, such as dentures, implants, and orthodontic devices, which provide surfaces for bacterial adherence; contaminated food, water, and behavioral habits, in which the oral route allows direct entry of gut or environmental bacteria; and oral–gastrointestinal complications, in which reflux facilitates the entry of gastric bacteria into the oral cavity.

- **Fecal-oral transmission** via contaminated food, unsafe drinking water, or poor hygiene can introduce pathogens such as *E. coli, Salmonella* spp., and *Shigella* spp., which may be transiently present and interact with the resident microbiota, increasing infection risk, especially in areas with poor sanitation [142].
- **Interpersonal contact,** including saliva exchange during kissing, contact with contaminated surfaces, animal contact, and behaviors such as nail‑biting or oral contact with unclean objects, particularly in children, facilitates microbial entry [6,101]. Moreover, sexual practices have been suggested as a transmission route for *H. pylori* to the oral cavity, and its transient presence on the tongue, cheeks, and palate, along with its ability to survive under both acidic and neutral pH conditions, indicates adaptability for extra‑gastric presence and transient oral colonization [143,144].
- **Reverse flow of gastric contents** through vomiting or gastroesophageal reflux can introduce gastric microorganisms into the mouth, contributing to tooth erosion and secondary infections, highlighting the microbial continuum between the oral cavity and the GI tract [145].
- **Familial microbial transmission** may contribute to the acquisition of the oral microbiota. While vertical transmission from mother to child is well established for indigenous oral bacteria, particularly *S. mutans* [146], recent strain-level metagenomic studies indicate that oral microbial transmission occurs predominantly through horizontal transmission among cohabiting individuals, with strain sharing increasing over time [147]. While prolonged household cohabitation could theoretically increase opportunities for microbial exchange, evidence for household transmission of non-oral and opportunistic bacteria remains limited, and longitudinal strain-level studies are needed to determine whether cohabitation contributes to their acquisition and persistent colonization [148].
- **Hematogenous spread** allows microorganisms from distant body sites to reach the mouth during systemic infections (bacteremia or sepsis), while conversely, oral microbes may disseminate through the bloodstream and contribute to systemic conditions such as infective endocarditis [149].
- **Aspiration of oropharyngeal secretions** is an important pathway, especially in intensive care units (ICUs) patients with neurological impairment, dysphagia, or those receiving mechanical ventilation, with pathogens such as *P. aeruginosa* and *K. pneumoniae* frequently detected in the oral cavity and associated with hospital‑acquired pneumonia [150].
- **Dental appliances**, including removable dentures, orthodontic devices, and implants, provide abiotic surfaces for microbial adhesion and biofilm formation, potentially harboring non‑oral and antibiotic‑resistant species, thereby increasing the risk of oral infections and systemic complications, especially in immunocompromised individuals [151].
- **Domesticated animals and pets,** including dogs and cats. Face-licking, kissing, sharing food or utensils, and exposure to saliva via bites or scratches can transiently lead to the introduction of animal-associated bacteria into the human oral cavity [152,153]. Although occasional cross-species transmission of canine-associated bacteria, including *Neisseria shayeganii, Porphyromonas canigingivalis, Tannerella forsythia, Streptococcus minor*, and *Helicobacter* spp., has been suggested, the prevalence of persistent colonization in humans has not been systematically reported. The highly specialized physicochemical environment of the oral cavity, together with host-specific ecological adaptation and microbial competition, is thought to limit the establishment of these microorganisms [154,155].
- **Factors facilitating persistence of non-oral bacteria** include poor oral hygiene, infrequent brushing, plaque accumulation, immune suppression, and neglected prosthesis care, which disrupt local defenses and enable exogenous pathogens to persist; patients undergoing chemotherapy, organ transplantation, or living with HIV are particularly vulnerable [156].

The relevance of each entry route differs by organism: fecal–oral transmission predominates for members of *Enterobacteriaceae* and *C. difficile*, aspiration for *P. aeruginosa* and *K. pneumoniae*, the gastro–oral route for *H. pylori*, and interpersonal contact for *S. aureus* and *A. baumannii*. Thus, colonization pathways are species-specific and depend on each organism's origin and physiological tolerance to oral conditions. Once introduced into the oral cavity, successful persistence depends on the ability of non-oral bacteria to adapt to their unique ecological environment.

### Microbial cooperation and adaptive traits

The stability and consistency of oral microbial communities, particularly at the genus level, have been well‑documented, providing a foundation for the complex interplay of microbial survival strategies within the oral cavity [157]. In this dynamic environment characterized by varying conditions and nutrient supply, microbial survival relies heavily on interspecies interactions, which can be either mutualistic or antagonistic. Mutualistic interactions, such as coaggregation and cell‑to‑cell adhesion, facilitate biofilm formation and enhance microbial persistence, while antagonistic interactions can prevent colonization by non‑resident species. Among oral bacteria, common coaggregation partners include *P. gingivalis, A. actinomycetemcomitans, E. faecalis* and *F. nucleatum*, all linked to periodontal diseases. Notably, *F. nucleatum* can co‑aggregate with non‑oral bacteria such as *H. pylori* and *S. aureus* through its surface protein RadD, as demonstrated *in vitro* [158], mediating interspecies adhesion and bridging early and late colonizers within biofilms; however, the *in vivo* relevance of these partnerships requires further investigation. Coaggregation is not limited to pathogenic consortia, as even non‑oral probiotics can co‑aggregate with key oral pathogens in a strain‑ and pathogen‑specific manner, which could be exploited to disrupt pathogenic biofilms and modulate disease‑associated microbial networks [159]. In addition to these cooperative strategies, non‑oral opportunistic pathogens also independently possess adaptive traits enabling persistence. *A. baumannii* exhibits multiple virulence factors, including lipocalin production, robust biofilm formation, siderophore‑mediated iron uptake, outer membrane protein A expression, and metabolic flexibility, allowing glucose‑independent growth [6]. Similarly, while *C. albicans* is often present at low abundance in healthy individuals; it can become clinically significant under conditions favoring overgrowth. During reduced immune response, *C. albicans* adheres strongly to epithelial cells via host transglutaminase binding to HWP1, and ALS3-mediated invasin activity with upregulated HWP1 expression further enhances biofilm formation, particularly on dentures, and contributes to increased virulence and antifungal resistance. Cooperative interactions with *S. gordonii* through ALS3‑SspB protein interaction further promote hyphal development and mixed‑species biofilms, augmenting its invasive potential [160].

### Entering viable-but-non- cultivable (VBNC) state

Bacterial dormancy states include various physiological states with reduced metabolism and stress tolerance, but these states are not identical. Persister cells are a small subpopulation that survives antibiotics and other forms of environmental stress via a transient metabolic slowdown while remaining cultivable. In contrast, VBNC cells lose the ability to form colonies on routine media, yet retain viability, membrane integrity, or gene expression, and can resume growth under appropriate resuscitation conditions [7]. Notably, direct evidence for the VBNC concept in clinical relevance in the oral cavity remains elusive. The VBNC state was first described by Xu et al. [161] in *Escherichia coli* and *Vibrio cholerae* as a survival response to stresses such as nutrient depletion and temperature shifts. McDougald et al. [162] later emphasized that its hallmark is a profound loss of cultivability, unlike other dormant forms, and proposed it as an adaptive strategy rather than a sign of cellular deterioration, since cells remain viable and can recover. Whether this state represents a genuine adaptive program or a step toward cell death is still debated, but these foundational studies established loss of cultivability as the defining feature that sets VBNC apart from general dormancy.

Transition into the VBNC state has been reported for several non‑oral pathogens detected in oral samples, including *H. pylori* [41], *E. faecalis* [163] and *S. aureus* [164], with *Campylobacter concisus* also described in this context [165]. However, the strength of evidence varies considerably; for *H. pylori* and *E. faecalis*, VBNC entry has been characterized in some detail, whereas for *K. pneumoniae,* evidence is largely inferred from non‑oral or environmental studies, and for *A. baumannii*, direct evidence remains even more limited. For *H. pylori* specifically, transition into a VBNC state under environmental stresses such as nutrient depletion, hypoxia, and microbial antagonism has been described [166,167]. Correlative light and electron microscopy recently provided the first ultrastructural visualization of coccoid and intermediate *H. pylori* forms in human saliva; however, this study did not directly assess bacterial viability or resuscitation [168]. Its contribution to recurrent gastric infection, therefore, remains speculative. Similarly, *E. faecalis* can adopt a VBNC state under nutrient-limited *in vitro* conditions [7], but whether this state contributes to persistent endodontic infections *in vivo* remains an open question. For *K. pneumoniae*, VBNC entry enables prolonged survival and evasion of detection by standard culture methods [169], though evidence for this mechanism, specifically within the oral cavity, remains largely inferred from non-oral or environmental studies.

Advanced techniques such as PMA‑qPCR, LIVE/DEAD staining, and flow cytometry have been used to detect putative VBNC cells in oral samples [7], but these approaches cannot fully distinguish a true VBNC reservoir from non‑viable or dying cells, and for most of the organisms discussed above, resuscitation to a cultivable, infectious phenotype has not been demonstrated directly from oral isolates. The clinical significance of VBNC detection in the oral cavity, therefore, remains an open question rather than an established mechanism of recurrent infection, and we treat it as such throughout this review.

### Biofilm formation

Biofilm formation is a key mechanism enabling non‑oral bacteria to adapt and persist in the oral cavity. These structured microbial communities, encased in an extracellular matrix, protect bacteria from immune defenses and antimicrobials by limiting immune cell access and reducing antibiotic penetration. Within biofilms, bacteria can enter low‑metabolic or dormant states, and environmental changes such as reduced oxygen tension, altered pH, and localized nutrient accumulation further enhance their survival [170]. Experimental evidence supports this adaptability. For instance, Thurnheer et al. demonstrated that *Escherichia coli*, *Staphylococcus aureus*, and *Enterococcus faecalis* can integrate into a six-species supragingival biofilm model [171]. While *A. actinomycetemcomitans* and *S. aureus* formed small aggregates within the biofilm mass, *E. faecalis* and *E. coli* grew vigorously, suppressing native species such as *S. mutans* and *A. oris,* illustrating that non‑oral and opportunistic pathogens can integrate into oral biofilms where they may be associated with dysbiosis and enhanced pathogenic potential. Periodontitis is characterized by a dysbiotic subgingival biofilm dominated by anaerobic bacteria [172]. However, facultative and non-oral bacteria, including multidrug‑resistant enterococci, staphylococci*, H. pylori,* and *members of* Pseudomonadales and Enterobacterales have also been detected within periodontal biofilms; their detection does not necessarily indicate that they are established periodontal pathogens, but rather suggests that the dysbiotic periodontal environment may permit their transient persistence or coexistence with the resident microbiota [133]. As a strict anaerobe, *C. difficile* can colonize dysbiotic subgingival biofilms [34], with molecular studies reporting high detection rates of *C. difficile*‑specific genes (*tpi, tcdA/tcdB*) in patients with severe periodontitis (>70% in diseased sites) correlating with inflammation, while toxin genes are absent in healthy individuals [34], TcdB disrupts epithelial integrity, induces tissue damage, and triggers pro‑inflammatory responses, increasing susceptibility to colonization particularly in inflammatory contexts such as periodontitis or inflammatory bowel disease [173].

Analogous to the gut ecosystem, these bacteria form complex biofilms with multiple microbial interactions that may inhibit, protect, or enhance virulence [174], and within these communities, *C. difficile* cells or spores are sheltered, potentially reducing treatment effectiveness and contributing to oral–fecal transmission. The strictly anaerobic and inflammatory environment of periodontal pockets further promotes interactions between *C. difficile* and dysbiotic microorganisms, such as *Fusobacterium* spp., *enterococci*, Gram‑negative bacilli, *Filifactor* spp., *Peptostreptococcus* spp., and *Veillonella* spp. [175]. Survival is also enhanced through interactions with *C. albicans* [176] and *F. nucleatum* [177], which co‑aggregate via adhesins and together stabilize biofilms, *F. nucleatum* additionally modulates host immunity, impairs neutrophil function, and produces metabolites such as putrescine that support biofilm integrity [178]. Adherence to early colonizers, particularly streptococci, facilitates *C. difficile* integration into the oral microbial community [179], with additional factors supporting persistence including favorable metabolites (bile acids, amino acids, succinate, sialic acids, tryptophan or indole catabolites), spore formation with low germination efficiency, adhesion to extracellular matrix proteins, and potential internalization into non‑phagocytic gingival cells [180,181].

### Niche selection

Each habitat within the human oral cavity, from the tooth surface and gingival sulcus to the tongue, cheek, and palate, provides consistently warm, moist, and nutrient-rich conditions with distinct environmental characteristics [6,171]. Key factors, including oxygen availability, nutrient sources (e.g. salivary mucins and gingival crevicular fluid), pH, and host immune interactions, shape microbial survival and colonization [182]. Ultimately, niche selection critically determines the fate of both resident and transient non-oral bacteria, reflecting strong selective pressures [183].

These non-resident bacteria reach the oral cavity via ingestion, aspiration, or systemic pathways, while diverse oral surfaces and variations in saliva flow may provide opportunities for their transient survival and establishment [184,185]. Following their entry, non-oral bacteria interact with the resident microbiota through cooperative and competitive mechanisms that can alter community structure and disrupt microbial homeostasis. Once dysbiosis is established, inflammation develops as a host defense response, initiating a self-reinforcing cycle that promotes the persistence of non-oral bacteria within dysbiotic communities [186,187]. Exposed tooth surfaces are more accessible to oxygen and may support aerobic or facultative anaerobic species such as *P. aeruginosa* and *A. baumannii*, whereas the gingival sulcus and periodontal pockets provide low-oxygen, nutrient-rich environments that may be permissive for gut-associated bacteria (e.g. *Klebsiella* spp. and *Serratia* spp.), *C. difficile*, and *H. pylori* [23,88]. However, direct *in vivo* evidence demonstrating active colonization of these niches by specific non-oral bacteria remains limited, and current knowledge is largely based on associative rather than mechanistic studies [188].

Inflammation within the gingival tissues is a major driver of niche selection. Increased gingival crevicular fluid supplies proteins and iron, while connective tissue breakdown releases collagen peptides and heme-containing compounds, transforming the subgingival environment into a nutrient-rich niche that selectively favors anaerobic and proteolytic bacteria, including some non-oral species keystone pathogens that further sustain this inflammatory state to preserve their nutrient supply, accelerating dysbiosis and tissue destruction [189,190]. Similar inflammatory niches may also facilitate the transient persistence or colonization of certain non-oral bacteria by providing conditions analogous to those exploited by oral inflammophiles [36].

Dental implants and natural teeth differ substantially in their biological, anatomical, and physicochemical characteristics, creating distinct ecological niches that influence microbial composition [191]. *Filifactor alocis*, a species frequently associated with periodontitis-associated dysbiotic biofilms [192,193], has also been reported at increased prevalence in peri-implantitis lesions [194]. In addition, bacteria such as *H. pylori* [195] and *Mycoplasma* spp. [196] have been detected around implants, with *H. pylori* showing a higher prevalence among patients with peri-implantitis and *Mycoplasma* spp. occurring at higher levels in peri-implant communities than in periodontitis-associated biofilms, suggesting that peri-implant inflammatory niches may support the transient persistence or colonization of non-oral bacteria of gastrointestinal or respiratory origin.

Beyond inflammation, antibiotic and antiseptic treatments can reshape the oral microbiome by creating vacant ecological niches [88], facilitating colonization by antimicrobial-resistant oral opportunists as well as exogenous non-oral bacteria with intrinsic or acquired resistance mechanisms [197]. Furthermore, some non-native bacteria entering the oral cavity (e.g. from dental unit waterlines) may establish themselves by integrating into existing biofilms through specific cell-to-cell interactions such as coaggregation [198].

### Clinical implications of non-oral bacteria

The oral cavity serves as both a local reservoir and a transient portal for non-oral bacteria. The primary concern with non-oral bacterial organisms is not their mere detection in healthy individuals, where they may represent transient passage, but rather their relative abundance and clinical significance in vulnerable populations, such as immunocompromised patients, those with periodontitis, or individuals with indwelling medical devices, where their pathogenic potential and biofilm persistence raise concerns. Beyond their direct pathogenic potential, non-oral bacteria may also contribute to antimicrobial resistance by serving as reservoirs of antibiotic resistance genes within multispecies oral biofilms (see Antibiotic Resistance Reservoir below). These microbes can enter the bloodstream during routine activities or dental procedures, particularly when mucosal integrity is disrupted, and may induce transient bacteremia, infective endocarditis (*Enterococcus* spp.), pulmonary infections (*K. pneumoniae* and *P. aeruginosa*), or adverse pregnancy outcomes [6]. Persistent colonization may also sustain low‑grade inflammation, damaging local tissues and exacerbating systemic conditions such as cardiovascular disease and diabetes [199]. Early recognition and monitoring are therefore essential for preventing infections, controlling antibiotic resistance, and promoting overall health.

#### Dissemination to bone and deep tissue

Beyond mucosal surfaces and dental biofilms, non-oral bacteria are also encountered in bone infections of the jaws, particularly in medication-related osteonecrosis of the jaw (MRONJ) and osteomyelitis. MRONJ, a serious adverse event following antiresorptive therapy, is characterized by exposed necrotic bone and chronic inflammation, where microbial colonization is increasingly recognized as a key pathogenic factor [200]. Traditional culture and molecular methods have revealed a highly heterogeneous microbiota in MRONJ lesions, with *Actinomyces* spp. and Actinomyces-like organisms being the most frequently detected, alongside *Fusobacterium*, *Prevotella*, Gram-positive anaerobic cocci, and *Streptococcus* spp. [201]. Importantly, non-oral bacteria such as *P. aeruginosa*, *E. faecalis*, *E. cloacae* and *K. pneumoniae* have also been isolated from osteonecrotic lesions, particularly in immunocompromised patients or those with chronic kidney disease [202,203]. The presence of microbial biofilms on necrotic bone surfaces, often composed of polymicrobial communities including both Gram-positive and Gram-negative organisms, further complicates the distinction between colonization and true infection [204]. Moreover, bisphosphonates may enhance bacterial adhesion to hydroxyapatite, potentially selecting for specific pathogens and promoting persistent colonization [205].

### Limitations in diagnostic and detection

Infectious diseases remain a leading cause of morbidity and mortality, particularly in elderly patients and those with chronic comorbidities, where delayed or inappropriate treatment can rapidly lead to severe complications such as sepsis and multi‑organ failure [206]. This clinical urgency is compounded in oral polymicrobial infections, where accurate diagnosis is challenging, especially when non‑oral species are present [207].

- **False-positive genetic results** arise from saliva containing non‑oral microbial DNA that can align atypically to the human reference genome, leading to incorrect genotype calls [208], and the presence of non‑human DNA can cause systems to mistakenly identify genetic variations in the human genome when these changes actually originate from microorganisms [209].
- **Misattribution to contamination** occurs because non‑oral bacteria present in saliva, dental plaque, or tissue samples can serve as reservoirs of antibiotic resistance genes and may be dismissed as contaminants when their clinical relevance is not recognized, even though they may represent true colonizers, which can complicate interpretation [158]. This risk is compounded when such bacteria enter a VBNC state, evading conventional culture-based detection (see VBNC section).
- **Overlooked opportunistic pathogens in vulnerable populations** are a concern in immunocompromised patients (e.g. those with end‑stage liver disease or undergoing chemotherapy), where non‑oral bacteria such as *E. coli, K. pneumoniae* and *P. aeruginosa* can overgrow due to suppression of the resident oral microbiota. Unlike fastidious oral anaerobes, these organisms grow well on standard laboratory media and are comparatively straightforward to culture, yet their presence may remain unrecognized because of limited clinical suspicion [210].
- **Limitations of conventional methods** include limited sensitivity for fastidious oral anaerobes [211]. Metagenomic next‑generation sequencing (mNGS) provides a comprehensive approach for pathogen detection, allowing the comparison of atypical oral microorganisms with potential source populations and the evaluation of host immune responses [104].
- **High background noise in molecular diagnostics** is caused by the high microbial diversity and the presence of transient or low‑abundance taxa that can obscure the distinction between true pathogens and background microbiota [212]. A WGS study showed that over 60% of anomalous reads in saliva are bacterial, and more than 80% of buccal swab and saliva samples contain >5% non‑human DNA, making it difficult to differentiate pathogens from background microbiota. Whether these non‑oral bacteria are true colonizers or merely part of the background microbiota acquired from the diet or environment remains unclear [209].
- **Environmental and procedural contamination** further complicate interpretation, as bacteria are detected on approximately 38% of dental clinic surfaces, and denture wearers often carry *E. coli* and *K. pneumoniae*, which may be misinterpreted as contamination rather than true colonization [213].
- **Laboratory variability** includes methodological inconsistencies across diagnostic laboratories that can further complicate interpretation, with insufficient quality control measures and a lack of standardized procedures affecting pathogen identification and antimicrobial susceptibility testing [214].

### Antibiotic resistance reservoir

The oral cavity constitutes a highly dynamic microbial ecosystem and a critical ecological interface between oral and extra-oral microbiomes. Colonization by non-oral bacteria introduces antibiotic resistance genes (ARGs) and mobile genetic elements into multispecies oral biofilms, where they may disseminate among resident bacteria through horizontal gene transfer (HGT), contributing to the oral resistome and the establishment of multidrug-resistant (MDR) reservoirs [85,215]. From a clinical and public health perspective, antimicrobial resistance (AMR) is a major global threat, responsible for 1.14 million direct and 4.71 million associated deaths in 2021, with projections indicating a marked increase by 2050. As previously noted, dental antibiotic prescribing combined with extensive antiseptic use, especially in sublethal concentrations for long periods, further increases selective pressure within the oral ecosystem [216]. Although current evidence does not support the modification of existing dental antibiotic prophylaxis protocols solely on the basis of detecting antimicrobial-resistant oral microorganisms, these findings underscore the importance of enhanced surveillance, risk assessment in vulnerable patients, and further clinical investigation.

Evidence supporting the oral cavity as a reservoir of ARGs derives from complementary genomic, metagenomic, and experimental studies. Genomic analyses have identified oral isolates of *E. cloacae* and *K. pneumoniae* harboring blaTEM, blaSHV, tetM and qnrB, with more than 90% exhibiting resistance to amoxicillin-clavulanic acid [217], while metagenomic studies demonstrate a substantial potential for HGT within the oral resistome [218]. *In vitro* studies further showed that conjugative plasmid transfer occurs between oral bacteria, including transfer from *E. faecalis* to oral *streptococci*, and that biofilm environments markedly enhance HGT compared with planktonic conditions [219,220]. Even low-abundance or transient bacteria may introduce and disseminate ARGs through HGT, as demonstrated by intergeneric transfer of the conjugative transposon Tn5397 from *Bacillus subtilis* to oral *Streptococcus* species within mixed-species biofilms [221]. Similarly, pheromone-mediated communication between oral *streptococci* and *E. faecalis* promotes conjugative plasmid transfer and dissemination of resistance genes among oral bacteria, contributing to the genetic plasticity of the oral metagenome [222]. Nevertheless, direct *in vivo* evidence demonstrating HGT within the human oral cavity remains limited. Current evidence supporting the oral cavity as a reservoir of antimicrobial resistance, therefore, includes:

- **High carriage of resistant *Enterobacteriaceae*** as described in detail above.
- **Elevated resistance in vulnerable populations** with oncology patients and individuals with CKD undergoing peritoneal dialysis exhibits higher colonization rates by multidrug‑resistant *Klebsiella, E. coli,* and *Raoultella* species compared with healthy individuals [223], and high rates of cephalosporin‑ and carbapenem‑resistant GNR colonization are also documented in healthy adolescents, including ESBL‑producing *Enterobacter, Klebsiella,* and *Serratia* species [224].
- **Oral cavity is an independent MRSA reservoir** with multidrug resistance more common in oral than nasal isolates (see *S. aureus* section above).
- **Antiseptics co-select for antibiotic resistance** as repeated exposure to CHX and cetylpyridinium chloride (CPC) does not eliminate biofilms but enriches resistant taxa with cross‑resistance to β‑lactams and clindamycin [88]. Though evidence remains insufficient to recommend abandoning CHX or CPC in routine dental practice, current strategies should emphasize targeted and short‑term use, adherence to antimicrobial stewardship, mechanical biofilm control, and monitoring of emerging resistance patterns.
- **Biofilm-driven resistance** with the EPS matrix acting as both a physical and chemical barrier, enhances antibiotic tolerance and enables horizontal gene transfer. In oral biofilms, *P. aeruginosa* and *E. faecalis* display multidrug resistance to penicillin while remaining susceptible to amoxicillin [73], and also exhibit resistance to tetracycline, ciprofloxacin, chloramphenicol, vancomycin and tigecycline [127].

Collectively, the oral cavity functions as a dynamic reservoir of antibiotic resistance, where dense multispecies biofilms and mobile genetic elements favor ARG maintenance and exchange via horizontal gene transfer among both non‑oral and resident bacteria. Strain‑resolved longitudinal studies, alongside surveillance and prudent antimicrobial use, are needed to limit resistance dissemination across mucosal ecosystems [225].

## Conclusion and future directions

The oral cavity should no longer be viewed solely as a closed ecosystem hosting a strictly defined resident microbiota, but rather as a dynamic interface where a diverse array of non‑oral bacteria, each with distinct physiological properties and colonization strategies, may transiently or persistently reside. Increasing evidence shows that enteric species such as Enterobacteriaceae, respiratory pathogens such as *P. aeruginosa*, and gastrointestinal organisms, including *C. difficile,* have been detected in oral samples. Advances in molecular technologies, particularly high‑throughput sequencing, have enabled the identification of these organisms, expanding our understanding of the oral microbiome beyond traditional culture‑based paradigms. Future research should focus on the development and optimization of protocols for the isolation and detection of non‑oral bacteria from dental biofilm and saliva samples. Key gaps remain in epidemiological, mechanistic, and pathogenic investigations. While culture‑based detection of non‑oral opportunistic bacteria is feasible under certain clinical conditions, a major challenge lies in distinguishing true colonization from transient contamination, especially in routine diagnostics where non‑oral bacteria may be dismissed as contaminants despite their pathogenic potential. From a pathogenesis perspective, studies should quantify spores, assess bacterial viability, measure toxin production or expression, and characterize predominant ribotypes, which distinguish genetically related bacterial strains based on ribosomal RNA gene polymorphisms. Epidemiologically, large‑scale, population‑based studies are necessary to determine the prevalence and distribution of non‑oral bacteria across diverse cohorts, including healthy individuals, patients with periodontal disease or peri‑implantitis, immunocompromised populations, and geographically isolated groups.

Non‑oral bacteria may also disseminate antibiotic resistance genes and virulence factors to resident oral strains, amplifying disease risk. Advanced experimental models and molecular tools, including 16S rRNA sequencing, metagenomics, and shotgun metagenomics, are essential for linking microbial composition with functional traits such as antibiotic resistance and virulence activity. *In vitro* multispecies biofilm models and *in vivo* systems can further clarify colonization mechanisms, interspecies interactions, immune evasion, and systemic consequences. Translating these insights into clinical practice will require robust diagnostics, rapid point‑of‑care assays, and the incorporation of oral microbiome profiling into personalized risk assessment. The diagnostic performance of rapid assays should be evaluated in oral samples from asymptomatic individuals, as well as patients with *C. difficile* infection, inflammatory bowel disease, pneumonia, and other clinical conditions. Recognizing non‑oral bacteria as functionally relevant members of the oral microbiome opens new therapeutic opportunities, particularly in periodontal disease management. Microorganisms such as *Klebsiella* spp., often dismissed as contaminants, may possess pathogenic potential; targeted investigation could enhance treatment strategies, support microbiome modulation approaches, and restore microbial homeostasis (e.g. probiotic‑based interventions). Reliable data remain limited, in part because non‑oral bacteria have historically been treated as contaminants rather than potential colonizers, and suitable animal models that replicate their persistence and behavior in the oral cavity are lacking. Addressing these gaps is essential to fully understand the oral cavity from a microbial‑ecological perspective as a dynamic microbial interface with implications for both oral and systemic health.

## Data Availability

The datasets used and/or analyzed during the current study are available from the corresponding author on reasonable request.

## References

[1] Bhuyan R , Mohanty JN , Das S , et al. Periodontitis and its inflammatory changes linked to various systemic diseases: a review of its underlying mechanisms. Biomedicines. 2022;10(10):2659. doi: 10.3390/biomedicines10102659 PMC9599402 36289921

[2] Baker JL . Illuminating the oral microbiome and its host interactions: recent advancements in omics and bioinformatics technologies in the context of oral microbiome research. FEMS Microbiol Rev. 2023;47(5):1–15. doi: 10.1093/femsre/fuad051 PMC10503653 37667515

[3] Giordano-Kelhoffer B , Lorca C , March Llanes J , et al. Oral microbiota, its equilibrium and implications in the pathophysiology of human diseases: a systematic review. Biomedicines. 2022;10(8):1803. doi: 10.3390/biomedicines10081803 PMC9405223 36009350

[4] Al-Ahmad A , Müller N , Wiedmann-Al-Ahmad M , et al. Endodontic and salivary isolates of enterococcus faecalis integrate into biofilm from human salivary bacteria cultivated in vitro. J Endod. 2009;35(7):986–991. doi: 10.1016/j.joen.2009.04.013 19567320

[5] Dong J , Li W , Wang Q , et al. Relationships between oral microecosystem and respiratory diseases. Front Mol Biosci. 2022;8:2021. doi: 10.3389/fmolb.2021.718222 PMC8767498 35071321

[6] Zaatout N . Presence of non-oral bacteria in the oral cavity. Arch Microbiol. 2021;203(6):2747–2760. doi: 10.1007/s00203-021-02300-y PMC8012020 33791834

[7] Mahdizade Ari M , Scholz KJ , Cieplik F , et al. Viable but non-cultivable state in oral microbiota: a critical review of an underexplored microbial survival strategy. Front Cell Infect Microbiol. 2025;15:2025. doi: 10.3389/fcimb.2025.1533768 PMC11959090 40171166

[8] Eriksen C , Boustedt K , Sonne SB , et al. Early life factors and oral microbial signatures define the risk of caries in a Swedish cohort of preschool children. NatSR. 2024;14(1):8463. doi: 10.1038/s41598-024-59126-z PMC11009336 38605085

[9] Spatafora G , Li Y , He X , et al. The evolving microbiome of dental caries. Microorganisms. 2024;12(1):121. doi: 10.3390/microorganisms12010121 PMC10819217 38257948

[10] Zhao H , Chu M , Huang Z , et al. Variations in oral microbiota associated with oral cancer. NatSR. 2017;7(1):11773. doi: 10.1038/s41598-017-11779-9 PMC5603520 28924229

[11] Burcham ZM , Garneau NL , Comstock SS , et al. Patterns of oral microbiota diversity in adults and children: a crowdsourced population study. NatSR. 2020;10(1):2133. doi: 10.1038/s41598-020-59016-0 PMC7005749 32034250

[12] Wolf M , Steinberg T , Scholz K , Kruse A , Rezasoltani S , Conrads G , Al-Ahmad A , Cieplik F . The rise and evolving role of fusobacterium nucleatum subspecies. Curr Res Microb Sci. 2025;9:100414. 10.1016/j.crmicr.2025.100414 PMC12178932 40547902

[13] Lamont RJ , Koo H , Hajishengallis G . The oral microbiota: dynamic communities and host interactions. Nat Rev Microbiol. 2018;16(12):745–759. doi: 10.1038/s41579-018-0089-x PMC6278837 30301974

[14] Lu M , Xuan S , Wang Z . Oral microbiota: a new view of body health. Food Sci Hum Wellness. 2019;8(1):8–15. doi: 10.1016/j.fshw.2018.12.001

[15] Zhang Y , Wang X , Li H , et al. Human oral microbiota and its modulation for oral health. Biomed Pharmacother. 2018;99:883–893. doi: 10.1016/j.biopha.2018.01.146 29710488

[16] Fadhil Ali Malaa S , Abd Ali Abd Aun Jwad B , Khalis Al-Masoudi H . Assessment of entamoeba gingivalis and trichomonas tenax in patients with chronic diseases and its correlation with some risk factors. Arch Razi Inst. 2022;77(1):87–93. 10.22092/ARI.2021.356549.1868 PMC9288630 35891719

[17] Tonoyan L , Chevalier M , Vincent-Bugnas S , et al. Detection of epstein-barr virus in periodontitis: a review of methodological approaches. Microorganisms. 2020;9(1):72. doi: 10.3390/microorganisms9010072 PMC7823867 33383930

[18] Müller M , Weber A , Tennert C , et al. Laboratory investigation of intra-tooth variability in microbial communities from clinically isolated root canal and filling material. Clin Oral Investig. 2026;30(9):419. doi: 10.1007/s00784-026-07108-y PMC13518401 42645617

[19] Santonocito S , Giudice A , Polizzi A , et al. A cross-talk between diet and the oral microbiome: balance of nutrition on inflammation and immune system’s response during periodontitis. Nutrients. 2022;14(12):2426. doi: 10.3390/nu14122426 PMC9227938 35745156

[20] Caselli E , Fabbri C , D’Accolti M , et al. Defining the oral microbiome by whole-genome sequencing and resistome analysis: the complexity of the healthy picture. BMC Microbiol. 2020;20(1):120. doi: 10.1186/s12866-020-01801-y PMC7236360 32423437

[21] Zelasko S , Swaney MH , Sandstrom S , et al. Upper respiratory microbial communities of healthy populations are shaped by niche and age. Microbiome. 2024;12(1):206. doi: 10.1186/s40168-024-01940-8 PMC11490146 39425237

[22] Flores GE , Caporaso JG , Henley JB , et al. Temporal variability is a personalized feature of the human microbiome. Genome Biol. 2014;15:1–13. doi: 10.1186/s13059-014-0531-y PMC4252997 25517225

[23] Jakubovics NS . Saliva as the sole nutritional source in the development of multispecies communities in dental plaque. Microbiol Spectr. 2015;3(3). doi: 10.1128/microbiolspec.MBP-0013-2014 26185065

[24] Anderson AC , Jonas D , Huber I , Karygianni L , Wölber J , Hellwig E , Arweiler N , Vach K , Wittmer A , Al-Ahmad A . Enterococcus faecalis from food, clinical specimens, and oral sites: prevalence of virulence factors in association with biofilm formation. Front Microbiol. 2015;6:1534. 10.3389/fmicb.2015.01534 PMC4707231 26793174

[25] Tian S , Ding T , Li H . Oral microbiome in human health and diseases. mLife. 2024;3(3):367–383. doi: 10.1002/mlf2.12136 PMC11442140 39359681

[26] Souto R , Andrade A , Uzeda M , et al. Prevalence of “non-oral” pathogenic bacteria in subgingival biofilm of subjects with chronic periodontitis. Braz J Microbiol. 2006;37:208–215. doi: 10.1590/S1517-83822006000300002

[27] Dani A . Colonization and infection. Cent European J Urol. 2014;67(1):86–87. 10.5173/ceju.2014.01.art19 PMC4074726 24982790

[28] Vidana R , Sullivan Å , Billström H , et al. Enterococcus faecalis infection in root canals - host-derived or exogenous source? Lett Appl Microbiol. 2011;52(2):109–115. doi: 10.1111/j.1472-765X.2010.02972.x 21155997

[29] Crobach MJT , Vernon JJ , Loo VG , et al. Understanding clostridium difficile colonization. Clin Microbiol Rev. 2018;31(2):1–29. doi: 10.1128/CMR.00021-17 PMC5967689 29540433

[30] Snell S , Evans B , White E , Hurlbert A . The prevalence and impact of transient species in ecological communities. Ecology. 2018;99:1825–1833. 10.1002/ecy.2398 29802772

[31] Rivas Caldas R , Le Gall F , Revert K , et al. Pseudomonas aeruginosa and periodontal pathogens in the oral cavity and lungs of cystic fibrosis patients: a case-control study. J Clin Microbiol. 2015;53(6):1898–1907. doi: 10.1128/JCM.00368-15 PMC4432057 25854483

[32] Wang M , Ding P , Qian Y , et al. Association between oral diseases and oral helicobacter pylori infection: a systematic review and meta-analysis. Clin Oral Investig. 2025;30:9. doi: 10.1007/s00784-025-06691-w 41372677

[33] Gaeta C , Marruganti C , Ali IAA , et al. The presence of enterococcus faecalis in saliva as a risk factor for endodontic infection. Front Cell Infect Microbiol. 2023;13:1061645. doi: 10.3389/fcimb.2023.1061645 PMC10118032 37091670

[34] Leite de Oliveira Rosa I , de Oliveira Ferreira E , Vieira Colombo AP . Molecular detection of toxigenic clostridioides difficile in subgingival biofilm of severe periodontitis. Anaerobe. 2025;93:102955. doi: 10.1016/j.anaerobe.2025.102955 40096907

[35] Chiusaroli L , Facchin G , Cozzolino C , et al. Outbreak of mucocutaneous candida infections in newborns: clinical implications, investigation, and control measures. Antimicrob Steward Healthc Epidemiol. 2026;6(1):e28. doi: 10.1017/ash.2025.10282 PMC12835949 41607691

[36] Hajishengallis G . Periodontitis: from microbial immune subversion to systemic inflammation. Nat Rev Immunol. 2015;15(1):30–44. doi: 10.1038/nri3785 PMC4276050 25534621

[37] van Winkelhoff AJ , Rurenga P , Wekema-Mulder GJ , et al. Non-oral gram-negative facultative rods in chronic periodontitis microbiota. Microb Pathog. 2016;94:117–122. doi: 10.1016/j.micpath.2016.01.020 26835659

[38] Mladenova I . Clinical relevance of helicobacter pylori infection. J Clin Med. 2021;10(16):3473. doi: 10.3390/jcm10163473 PMC8396975 34441769

[39] Ranjbar R , Behzadi P , Farshad S . Advances in diagnosis and treatment of helicobacter pylori infection. Acta Microbiol Immunol Hung. 2017;64(3):273–292. doi: 10.1556/030.64.2017.008 28263101

[40] Burucoa C , Axon A . Epidemiology of Helicobacter pylori infection. Helicobacter. 2017;22(Suppl 1):1–5. doi: 10.1111/hel.12403 28891138

[41] Mao X , Jakubovics NS , Bächle M , et al. Colonization of helicobacter pylori in the oral cavity - an endless controversy? Crit Rev Microbiol. 2021;47(5):612–629. doi: 10.1080/1040841X.2021.1907740 33899666

[42] Zhao Y , Gao X , Guo J , Yu D , Xiao Y , Wang H , Li Y . Helicobacter pylori infection alters gastric and tongue coating microbial communities . Helicobacter. 2019;24(2):e12567. 10.1111/hel.12567 PMC6593728 30734438

[43] Irani S , Esfahani AM , Zerehpoush FB . Detection of helicobacter pylori in oral lesions. J Dent Res Dent Clin Dent Prospects. 2013;7(4):230–237. 10.5681/joddd.2013.037 PMC3935555 24578822

[44] Navabi N , Aramon M , Mirzazadeh A . Does the presence of the helicobacter pylori in the dental plaque associate with its gastric infection? A meta-analysis and systematic review. Dent Res J (Isfahan). 2011;8(4):178–182. doi: 10.4103/1735-3327.86033 PMC3221084 22135688

[45] Román-Román A , Giono-Cerezo S , Camorlinga-Ponce M , et al. vacA genotypes of helicobacter pylori in the oral cavity and stomach of patients with chronic gastritis and gastric ulcer. Enferm Infecc Microbiol Clin. 2013;31(3):130–135. doi: 10.1016/j.eimc.2012.09.002 23084752

[46] Ji Y , Liang X , Lu H . Analysis of by high-throughput sequencing: helicobacter pylori infection and salivary microbiome. BMC Oral Health. 2020;20(1):84. doi: 10.1186/s12903-020-01070-1 PMC7333272 32197614

[47] Somarathna MT , Leuke Bandara D , Gunasekare SKV , et al. Molecular detection of helicobacter pylori in saliva of Sri Lankan adults with periodontitis, gastritis or both conditions. BMC Oral Health. 2026;26(1):1076. doi: 10.1186/s12903-026-08421-4 PMC13281471 42032553

[48] Straka M , Borecová P , Csóková K , Gerža E , James I.J . Periodontitis, oral helicobacter pylori reservoirs, and gastric cancer: implications for adjunctive periodontal therapy. Neuro Endocrinol Lett. 2026;47:53–63. 41915928

[49] Moradi Y , Majidi L , Khateri S , et al. The association between periodontal diseases and helicobacter pylori: an updated meta-analysis of observational studies. BMC Oral Health. 2023;23(1):523. doi: 10.1186/s12903-023-03232-3 PMC10369707 37496045

[50] Scholz KJ , Höhne A , Wittmer A , et al. Co-culture of helicobacter pylori with oral microorganisms in human saliva. Clin Oral Investig. 2025;29(1):79. doi: 10.1007/s00784-025-06160-4 PMC11757641 39849235

[51] Zhang L , Chen X , Ren B , et al. Helicobacter pylori in the oral cavity: current evidence and potential survival strategies. Int J Mol Sci. 2022;23(21):13646. doi: 10.3390/ijms232113646 PMC9657019 36362445

[52] Khosravi Y , Dieye Y , Loke MF , et al. Streptococcus mitis induces conversion of helicobacter pylori to coccoid cells during co-culture in vitro. PLoS One. 2014;9(11):e112214. doi: 10.1371/journal.pone.0112214 PMC4227722 25386948

[53] Olbermann P , Josenhans C , Moodley Y , et al. A global overview of the genetic and functional diversity in the helicobacter pylori cag pathogenicity island. PLoS Genet. 2010;6(8):e1001069. doi: 10.1371/journal.pgen.1001069 PMC2924317 20808891

[54] Scott DR , Marcus EA , Weeks DL , Sachs G . Mechanisms of acid resistance due to the urease system of helicobacter pylori . Gastroenterology. 2002;123(1):187–195. 10.1053/gast.2002.34218 12105847

[55] Palencia SL , García A , Palencia M . Multiple surface interaction mechanisms direct the anchoring, co-aggregation and formation of dual-species biofilm between candida albicans and helicobacter pylori. J Adv Res. 2022;35:169–185. doi: 10.1016/j.jare.2021.03.013 PMC8721356 35024198

[56] Schmidinger B , Petri K , Lettl C , et al. Helicobacter pylori binds human annexins via lipopolysaccharide to interfere with toll-like receptor 4 signaling. PLoS Pathog. 2022;18(2):e1010326. doi: 10.1371/journal.ppat.1010326 PMC8890734 35176125

[57] Chen X , Zhou X , Liao B , et al. The cross-kingdom interaction between helicobacter pylori and candida albicans. PLoS Pathog. 2021;17(5):e1009515. doi: 10.1371/journal.ppat.1009515 PMC8101746 33956895

[58] Cheok YY , Tan GMY , Lee CYQ , et al. Innate immunity crosstalk with helicobacter pylori: pattern recognition receptors and cellular responses. Int J Mol Sci. 2022;23(14):7561. doi: 10.3390/ijms23147561 PMC9317022 35886908

[59] Bonsor DA , Zhao Q , Schmidinger B , et al. The helicobacter pylori adhesin protein HopQ exploits the dimer interface of human CEACAMs to facilitate translocation of the oncoprotein CagA. EMBO J. 2018;37(13):1–17. doi: 10.15252/embj.201798664 PMC6028028 29724755

[60] Thi MTT , Wibowo D , Rehm BHA . Pseudomonas aeruginosa biofilms. Int J Mol Sci. 2020;21(22):8671. doi: 10.3390/ijms21228671 PMC7698413 33212950

[61] Schwartz B , Klamer K , Zimmerman J , et al. Multidrug resistant pseudomonas aeruginosa in clinical settings: a review of resistance mechanisms and treatment strategies. Pathogens. 2024;13(11):975. doi: 10.3390/pathogens13110975 PMC11597786 39599528

[62] Komiyama K , Tynan JJ , Habbick BF , et al. Pseudomonas aeruginosa in the oral cavity and sputum of patients with cystic fibrosis. Oral Surg Oral Med Oral Pathol. 1985;59(6):590–594. doi: 10.1016/0030-4220(85)90187-2 3925406

[63] Bodey GP , Bolivar R , Fainstein V , et al. Infections caused by pseudomonas aeruginosa. Rev Infect Dis. 1983;5(2):279–313. doi: 10.1093/clinids/5.2.279 6405475

[64] Gordon N , Segal LN , Wu BG . Rethinking pneumonia in terms of the lung microbiome. Curr Opin Pulm Med. 2026;32(3):169–181. doi: 10.1097/MCP.0000000000001247 41631341

[65] Bellissimo-Rodrigues FB-RWT . Ventilator-associated pneumonia and oral health. Rev Soc Bras Med Trop. 2012;45(5):543–544. doi: 10.1590/S0037-86822012000500001 23152333

[66] O‘Donnell MJ , Boyle MA , Russell RJ , et al. Management of dental unit waterline biofilms in the 21st century. Future Microbiol. 2011;6(10):1209–1226. doi: 10.2217/fmb.11.104 22004039

[67] Baudet A , et al. Initial waterline contamination by pseudomonas aeruginosa in newly installed dental chairs . Microbiol Spectr. 2024;12(6):e0396223. 10.1128/spectrum.03962-23 PMC11237434 38652098

[68] Ouellet MM , Leduc A , Nadeau C , et al. Pseudomonas aeruginosa isolates from dental unit waterlines can be divided in two distinct groups, including one displaying phenotypes similar to isolates from cystic fibrosis patients. Front Microbiol. 2015;5:2014. doi: 10.3389/fmicb.2014.00802 PMC4301018 25653647

[69] Perez-Tanoira R , Aarnisalo A , Haapaniemi A , et al. Bacterial biofilm in salivary stones. Eur Arch Otorhinolaryngol. 2019;276(6):1815–1822. doi: 10.1007/s00405-019-05445-1 PMC6529597 31028534

[70] Vestby LK , Grønseth T , Simm R , et al. Bacterial biofilm and its role in the pathogenesis of disease. Antibiotics (Basel). 2020;9(2):59. doi: 10.3390/antibiotics9020059 PMC7167820 32028684

[71] De Grandi R , Capaccio P , Bidossi A , et al. Salivary calculi microbiota: new insights into microbial networks and pathogens reservoir. Microb Infect. 2018;21:109–112. doi: 10.1016/j.micinf.2018.10.002 30385304

[72] Zhang ZL , Qian KL , Xiao MZ . Global burden of lower respiratory infections associated with antimicrobial resistance: insights from the global burden of disease 2021. Antimicrob Resist Infect Control. 2026;15(1):45. doi: 10.1186/s13756-026-01720-z PMC13032516 41723555

[73] Wu X , Al Farraj DA , Rajaselvam J , et al. Characterization of biofilm formed by multidrug resistant pseudomonas aeruginosa DC-17 isolated from dental caries. Saudi J Biol Sci. 2020;27(11):2955–2960. doi: 10.1016/j.sjbs.2020.07.020 PMC7569125 33100852

[74] Bertolini M , Costa R , Barão V , et al. Oral microorganisms and biofilms: new insights to defeat the main etiologic factor of oral diseases. Microorganisms. 2022;10(12):2413. doi: 10.3390/microorganisms10122413 PMC9781395 36557666

[75] Bengoechea JA , Sa Pessoa J . Klebsiella pneumoniae infection biology: living to counteract host defences. FEMS Microbiol Rev. 2019;43(2):123–144. doi: 10.1093/femsre/fuy043 PMC6435446 30452654

[76] Zenati K , Braun SD , Belhadi D , et al. The oral cavity as a reservoir for resistance- and hypervirulence-associated genes of klebsiella pneumoniae in hospitalized patients. Front Microbiol. 2026;17:1751947. doi: 10.3389/fmicb.2026.1751947 PMC12916564 41725809

[77] Wyres KL , Holt KE . Klebsiella pneumoniae as a key trafficker of drug resistance genes from environmental to clinically important bacteria. Curr Opin Microbiol. 2018;45:131–139. doi: 10.1016/j.mib.2018.04.004 29723841

[78] Kaur CP , Vadivelu J , Chandramathi S . Impact of klebsiella pneumoniae in lower gastrointestinal tract diseases. J Dig Dis. 2018;19(5):262–271. doi: 10.1111/1751-2980.12595 29573336

[79] Bakti R , Pradnyani IGAS , Subarnbhesaj A , et al. Presence of klebsiella pneumoniae on black hairy tongue as oral microbiome dysbiosis. Interdental J Kedokt Gigi. 2025;21:331–334. doi: 10.46862/interdental.v21i2.12176

[80] Espíndola LCP , Picão RC , Mançano SMCN , et al. Prevalence and antimicrobial susceptibility of gram-negative bacilli in subgingival biofilm associated with periodontal diseases. J Periodontol. 2022;93(1):69–79. doi: 10.1002/JPER.20-0829 33955542

[81] Veras EL , Castro dos Santos N , Souza JGS , et al. Newly identified pathogens in periodontitis: evidence from an association and an elimination study. J Oral Microbiol. 2023;15(1):2213111. doi: 10.1080/20002297.2023.2213111 PMC10228317 37261036

[82] Kitamoto S , Nagao-Kitamoto H , Jiao Y , et al. The intermucosal connection between the mouth and gut in commensal pathobiont-driven colitis. Cell. 2020;182(2):447–462.e14. doi: 10.1016/j.cell.2020.05.048 PMC7414097 32758418

[83] Jepsen K , Falk W , Brune F , et al. Prevalence and antibiotic susceptibility trends of selected enterobacteriaceae, enterococci, and candida albicans in the subgingival microbiota of German periodontitis patients: a retrospective surveillance study. Antibiotics (Basel). 2022;11(3):385. doi: 10.3390/antibiotics11030385 PMC8944811 35326848

[84] Constantinides B , Chau KK , Quan TP , et al. Genomic surveillance of escherichia coli and klebsiella spp. In hospital sink drains and patients. Microb Genom. 2020;6(7):4–16. doi: 10.1099/mgen.0.000391 PMC7478627 32553019

[85] Liu J , Spencer N , Utter DR , et al. Persistent enrichment of multidrug-resistant klebsiella in oral and nasal communities during long-term starvation. Microbiome. 2024;12(1):132. doi: 10.1186/s40168-024-01854-5 PMC11264962 39030586

[86] Folliero V , Franci G , Dell'Annunziata F , Giugliano R , Foglia F , Sperlongano R , De Filippis A , Finamore E , Galdiero M . Evaluation of antibiotic resistance and biofilm production among clinical strain isolated from medical devices. Int J Microbiol. 2021;2021:9033278. 10.1155/2021/9033278 PMC8380152 34426740

[87] Jiang S , Zeng J , Zhou X , et al. Drug resistance and gene transfer mechanisms in Respiratory/Oral bacteria. J Dent Res. 2018;97(10):1092–1099. doi: 10.1177/0022034518782659 29928825

[88] Mao X , Hiergeist A , Auer DL , et al. Ecological effects of daily antiseptic treatment on microbial composition of saliva-grown microcosm biofilms and selection of resistant phenotypes. Front Microbiol. 2022;13:934525. doi: 10.3389/fmicb.2022.934525 PMC9280182 35847089

[89] Almatroudi A , Hu H , Deva A , et al. A new dry-surface biofilm model: an essential tool for efficacy testing of hospital surface decontamination procedures. J Microbiol Methods. 2015;117:171–176. doi: 10.1016/j.mimet.2015.08.003 26260119

[90] Sanmartín Zhunio BE , Monge Puma BM , Rodríguez Coyago ML . Oral and oropharyngeal colonization by enterobacteriaceae: a narrative review on its epidemiology and clinical impact. Medwave. 2025;25(3):e2938. doi: 10.5867/medwave.2025.03.2938 40198888

[91] Mirpuri J , Brazil JC , Berardinelli AJ , et al. Commensal escherichia coli reduces epithelial apoptosis through IFN-alphaA-mediated induction of guanylate binding protein-1 in human and murine models of developing intestine. J Immunol. 2010;184(12):7186–7195. doi: 10.4049/jimmunol.0903116 PMC3145319 20483731

[92] Mazurek-Popczyk J , Pisarska J , Bok E , et al. Antibacterial activity of bacteriocinogenic commensal escherichia coli against zoonotic strains resistant and sensitive to antibiotics. Antibiotics (Basel). 2020;9(7):411. doi: 10.3390/antibiotics9070411 PMC7400030 32679778

[93] Min H , Baek K , Lee A , et al. Genomic characterization of four escherichia coli strains isolated from oral lichen planus biopsies. J Oral Microbiol. 2021;13(1):1905958. doi: 10.1080/20002297.2021.1905958 PMC8009128 33828821

[94] Dashper SG , Nastri A , Abbott PV . *odontogenic bacterial infections* In: Farah CS , Balasubramaniam R , McCullough MJ , editors. Contemporary Oral Medicine: A Comprehensive Approach to Clinical Practice. Cham: Springer International Publishing; 2019. p. 819–870.

[95] Alghamdi S . Isolation and identification of the oral bacteria and their characterization for bacteriocin production in the oral cavity. Saudi J Biol Sci. 2022;29(1):318–323. doi: 10.1016/j.sjbs.2021.08.096 PMC8716906 35002424

[96] Wang X , Zhao Z , Tang N , et al. Microbial community analysis of saliva and biopsies in patients with oral lichen planus. Front Microbiol. 2020;11:629. doi: 10.3389/fmicb.2020.00629 PMC7219021 32435231

[97] Rasko DA , Rosovitz MJ , Myers GSA , et al. The pangenome structure of escherichia coli: comparative genomic analysis of E. Coli commensal and pathogenic isolates. J Bacteriol. 2008;190(20):6881–6893. doi: 10.1128/JB.00619-08 PMC2566221 18676672

[98] Brito-Aragão M , Gomes F , Rocha F , Pinto V , Barbosa F . Prevalence and susceptibility of enterobacteriaceae isolated from the saliva of students from the northeast of Brazil. Glob J Med Res. 2016;16(2):13–17.

[99] Back-Brito GN , El Ackhar VNR , Querido SMR , et al. Staphylococcus spp., enterobacteriaceae and pseudomonadaceae oral isolates from Brazilian HIV-positive patients. Correlation with CD4 cell counts and viral load. Arch Oral Biol. 2011;56(10):1041–1046. doi: 10.1016/j.archoralbio.2011.02.016 21420663

[100] Baek K , Lee J , Yoon H , et al. Characterization of intratissue bacterial communities and isolation of escherichia coli from oral lichen planus lesions. Sci Rep. 2020;10(1):3495. doi: 10.1038/s41598-020-60449-w PMC7044275 32103089

[101] Chinnasamy A , Ramalingam K , Chopra P , et al. Chronic nail biting, orthodontic treatment and enterobacteriaceae in the oral cavity . J Clin Exp Dent. 2019;11(12):e1157–e1162. 10.4317/jced.56059 PMC6894907 31824597

[102] Pathak AK , Sharma DS . Biofilm associated microorganisms on removable oral orthodontic appliances in children in the mixed dentition. J Clin Pediatr Dent. 2013;37(3):335–339. doi: 10.17796/jcpd.37.3.92230h6256v8697t 23855182

[103] Li L , Zhang Y , Liu X , et al. Periodontitis exacerbates and promotes the progression of chronic kidney disease through oral flora, cytokines, and oxidative stress. Front Microbiol. 2021;12:656372. doi: 10.3389/fmicb.2021.656372 PMC8238692 34211440

[104] Costa CF , Merino-Ribas A , Ferreira C , et al. Characterization of oral enterobacteriaceae prevalence and resistance profile in chronic kidney disease patients undergoing peritoneal dialysis. Front Microbiol. 2021;12:736685. doi: 10.3389/fmicb.2021.736685 PMC8713742 34970231

[105] Campos J , Pires MF , Sousa M , et al. Unveiling the relevance of the oral cavity as a staphylococcus aureus colonization site and potential source of antimicrobial resistance. Pathogens. 2023;12(6):765. doi: 10.3390/pathogens12060765 PMC10304336 37375455

[106] Murdoch FE , Sammons RL , Chapple IL . Isolation and characterization of subgingival staphylococci from periodontitis patients and controls. Oral Dis. 2004;10(3):155–162. doi: 10.1046/j.1601-0825.2003.01000.x 15089925

[107] Innes N , Johnson I , Al-Yaseen W , et al. A systematic review of droplet and aerosol generation in dentistry. J Dent. 2021;105:103556. doi: 10.1016/j.jdent.2020.103556 PMC7834118 33359043

[108] Ohara-Nemoto Y , Haraga H , Kimura S , et al. Occurrence of staphylococci in the oral cavities of healthy adults and nasal oral trafficking of the bacteria. J Med Microbiol. 2008;57(Pt 1):95–99. doi: 10.1099/jmm.0.47561-0 18065673

[109] Tawara Y , Honma K , Naito Y . Methicillin-resistant staphylococcus aureus and candida albicans on denture surfaces. Bull Tokyo Dent Coll. 1996;37(3):119–128. 9151568

[110] Quiros-Roldan E , Sottini A , Natali PG , et al. The impact of immune system aging on infectious diseases. Microorganisms. 2024;12(4):775. doi: 10.3390/microorganisms12040775 PMC11051847 38674719

[111] Santacroce L , Passarelli PC , Azzolino D , et al. Oral microbiota in human health and disease: a perspective. Exp Biol Med (Maywood). 2023;248(15):1288–1301. doi: 10.1177/15353702231187645 PMC10625343 37688509

[112] Donkor ES , Kotey FC . Methicillin-resistant staphylococcus aureus in the oral cavity: implications for antibiotic prophylaxis and surveillance. Infect Dis (Auckl). 2020;13:1178633720976581. doi: 10.1177/1178633720976581 PMC7739134 33402829

[113] Alam M , Bastakoti B . Therapeutic guidelines: antibiotic. Version 15. Aust Prescr. 2015;38(4):137–137. doi: 10.18773/austprescr.2015.049

[114] Cimolai N . The role of healthcare personnel in the maintenance and spread of methicillin-resistant staphylococcus aureus. J Infect Public Health. 2008;1(2):78–100. doi: 10.1016/j.jiph.2008.10.001 20701849

[115] Chmielewski M , Załachowska O , Komandera D , et al. The oral cavity-another reservoir of antimicrobial-resistant staphylococcus aureus? Antibiotics (Basel). 2024;13(7):649. doi: 10.3390/antibiotics13070649 PMC11273406 39061331

[116] Vanzato Palazzo IC , Gir E , Pimenta F , et al. Does the oral cavity represent an important reservoir for MRSA in healthcare workers? J Hosp Infect. 2010;76(3):277–278. doi: 10.1016/j.jhin.2010.04.018 20621393

[117] Zhu B , Macleod LC , Kitten T , et al. Streptococcus sanguinis biofilm formation & interaction with oral pathogens. Future Microbiol. 2018;13(8):915–932. doi: 10.2217/fmb-2018-0043 PMC6060398 29882414

[118] Gedefie A , Demsiss W , Belete MA , et al. Acinetobacter baumannii biofilm formation and its role in disease pathogenesis: a review. Infect Drug Resist. 2021;14:3711–3719. doi: 10.2147/IDR.S332051 PMC8439624 34531666

[119] Carruthers MD , Nicholson PA , Tracy EN , et al. Acinetobacter baumannii utilizes a type VI secretion system for bacterial competition. PLoS One. 2013;8(3):e59388. doi: 10.1371/journal.pone.0059388 PMC3602014 23527179

[120] Richards AM , Abu Kwaik Y , Lamont RJ . Code blue: acinetobacter baumannii, a nosocomial pathogen with a role in the oral cavity. Mol Oral Microbiol. 2015;30(1):2–15. doi: 10.1111/omi.12072 PMC4617346 25052812

[121] Bhargava N , Sharma P , Capalash N . N-acyl homoserine lactone mediated interspecies interactions between A. Baumannii and P. Aeruginosa. Biofouling. 2012;28(8):813–822. doi: 10.1080/08927014.2012.714372 22867087

[122] Sharma J , Jhamb S , Mehta M , et al. Characterization of enterococcus faecalis associated with root canal failures: virulence and resistance profile. J Conserv Dent Endod. 2025;28(7):602–606. doi: 10.4103/JCDE.JCDE_190_25 PMC12310113 40746465

[123] Komiyama EY , Lepesqueur LSS , Yassuda CG , et al. Enterococcus species in the oral cavity: prevalence, virulence factors and antimicrobial susceptibility. PLoS One. 2016;11(9):e0163001. doi: 10.1371/journal.pone.0163001 PMC5025163 27631785

[124] Al-Ahmad A , Maier J , Follo M , et al. Food-borne enterococci integrate into oral biofilm: an in vivo study. J Endod. 2010;36:1812–1819. doi: 10.1016/j.joen.2010.08.011 20951293

[125] Kaittan ZQ , Zghair ZR . Enterococcus spp from the oral cavity and wounds of slaughterhouse workers in Baghdad city. Journal of Medicinal and Pharmaceutical Chemistry Research. 2025;7(6):1174–1186.

[126] Khuda F , Jayusman P , Baharin B , Mohamad Anuar NN , Sharma A , Nasruddin N . Oral inoculation of enterococcus faecalis, DNA quantification and histopathological evaluation of gingival, heart and kidney tissue samples in rats. Iran J Microbiol. 2024;16(3):337–341. 10.18502/ijm.v16i3.15765 PMC11245354 39005598

[127] Parga A , Mattu J , Belibasakis GN , et al. A polymicrobial perspective into the ecological role of enterococcus faecalis in dental root canal infections. NPJ Biofilms Microbiomes. 2025;11(1):83. doi: 10.1038/s41522-025-00722-w PMC12098920 40404644

[128] Hossainpour H , Nazari Z , Moradi P , Vahedian N , Alvandi A , Khazaei S , Mahmoudi H . Enterococcus faecalis in untreated roots canal teeth, a microbiological study . Avicenna J Dent Res. 1–5. In press 2026

[129] Halkai R , Hegde M , Halkai K . Enterococcus faecalis can survive extreme challenges -Overview 1 2 3. Enterococcus Faecalis can Survive Extreme Challenges - Overviewnujhs. 2012;2(3):49–53 ISSN 2249-7110.

[130] Xu Z , Ma H , Jiang X , et al. Transcriptome analysis of calcium hydroxide tolerance in enterococcus faecalis. Front Microbiol. 2025;16:2025. doi: 10.3389/fmicb.2025.1551824 PMC12075564 40371114

[131] Chilton CH , Viprey V , Normington C , et al. Clostridioides difficile pathogenesis and control. Nat Rev Microbiol. 2025;24:215–232. doi: 10.1038/s41579-025-01242-2 41039149

[132] Dewhirst FE , Chen T , Izard J , et al. The human oral microbiome. J Bacteriol. 2010;192(19):5002–5017. doi: 10.1128/JB.00542-10 PMC2944498 20656903

[133] Henriques LC , de Brito LCN , Tavares WLF , et al. Microbial ecosystem analysis in root canal infections refractory to endodontic treatment. J Endod. 2016;42(8):1239–1245. doi: 10.1016/j.joen.2016.05.014 PMC5168558 27377440

[134] Wang L , Li D , Chen Z , et al. An atypical case of monomicrobial clostridioides difficile septicemia with no gastrointestinal manifestations. Front Cell Infect Microbiol. 2022;12:853252. doi: 10.3389/fcimb.2022.853252 PMC9009443 35433499

[135] Silva-Senem MX .e., Silva‐Senem M , Heller D , et al. Clinical and microbiological effects of systemic antimicrobials combined to an anti-infective mechanical debridement for the management of aggressive periodontitis: a 12-month randomized controlled trial. J Clin Periodontol. 2013;40(3):242–251. doi: 10.1111/jcpe.12052 23297772

[136] de Oliveira AM , Lourenço TGB , Colombo APV . Impact of systemic probiotics as adjuncts to subgingival instrumentation on the oral-gut microbiota associated with periodontitis: a randomized controlled clinical trial. J Periodontol. 2022;93(1):31–44. doi: 10.1002/JPER.21-0078 34028826

[137] Carlsen DB , Durkin MJ , Gibson G , et al. Concordance of antibiotic prescribing with the American dental association acute oral infection guidelines within Veterans' affairs (VA) dentistry. Infect Control Hosp Epidemiol. 2021;42(12):1422–1430. doi: 10.1017/ice.2021.16 PMC8410877 33650474

[138] Wilson GM , Evans CT , Fitzpatrick MA , et al. Clostridioides difficile infection following dental antibiotic prescriptions in a cohort of US veterans. Infect Control Hosp Epidemiol. 2023;44(3):494–496. doi: 10.1017/ice.2021.516 PMC9677728 35188097

[139] Metzger Z , Blasbalg J , Dotan M , et al. Enhanced attachment of porphyromonas gingivalis to human fibroblasts mediated by fusobacterium nucleatum. J Endod. 2009;35(1):82–85. doi: 10.1016/j.joen.2008.10.011 19084131

[140] Abranches J , Zeng L , Kajfasz JK , et al. Biology of oral streptococci. Microbiol Spectr. 2018;6(5):1–12. doi: 10.1128/microbiolspec.GPP3-0042-2018 PMC6287261 30338752

[141] Sharma N , Bhatia S , Singh Sodhi A , et al. Oral microbiome and health. AIMS Microbiol. 2018;4(1):42–66. doi: 10.3934/microbiol.2018.1.42 PMC6605021 31294203

[142] Sedghi L , DiMassa V , Harrington A , et al. The oral microbiome: role of key organisms and complex networks in oral health and disease. Periodontol 2000. 2021;87(1):107–131. doi: 10.1111/prd.12393 PMC8457218 34463991

[143] Kort R , Caspers M , van de Graaf A , et al. Shaping the oral microbiota through intimate kissing. Microbiome. 2014;2:41. doi: 10.1186/2049-2618-2-41 PMC4233210 25408893

[144] Dimitriadi D . Helicobacter pylori: a sexually transmitted bacterium? Cent European J Urol. 2014;67(4):407–409. doi: 10.5173/ceju.2014.04.art18 PMC4310890 25667764

[145] Huttenhower C , Gevers D , Knight R , Abubucker S , Badger JH , Chinwalla AT . Structure, function and diversity of the healthy human microbiome. Nature. 2012;486(7402):207–214. doi: 10.1038/nature11234 PMC3564958 22699609

[146] Bastos V , da Silva Bastos V , Freitas-Fernandes LB , et al. Mother-to-child transmission of streptococcus mutans: a systematic review and meta-analysis. J Dent. 2015;43:181–191. doi: 10.1016/j.jdent.2014.12.001 25486222

[147] Valles-Colomer M , Blanco-Míguez A , Manghi P , et al. The person-to-person transmission landscape of the gut and oral microbiomes. Nature. 2023;614(7946):125–135. doi: 10.1038/s41586-022-05620-1 PMC9892008 36653448

[148] Azevedo MJ , Garcia A , Costa CF , et al. The contribution of maternal factors to the oral microbiota of the child: influence from early life and clinical relevance. Jpn Dent Sci Rev. 2023;59:191–202. doi: 10.1016/j.jdsr.2023.06.002 PMC10320028 37415593

[149] Gendron R , Grenier D , Maheu-Robert L-F . The oral cavity as a reservoir of bacterial pathogens for focal infections. Microb Infect. 2000;2(8):897–906. doi: 10.1016/S1286-4579(00)00391-9 10962273

[150] Winning L , Lundy FT , Blackwood B , et al. Oral health care for the critically ill: a narrative review. Crit Care. 2021;25(1):353. doi: 10.1186/s13054-021-03765-5 PMC8485109 34598718

[151] Rimondini L , Cochis A , Varoni E , et al. Biofilm formation on implants and prosthetic dental materials In: Handbook of bioceramics and biocomposites. Springer; 2016. p. 991–1027. doi: 10.1007/978-3-319-12460-5_48

[152] Podar NA , Carrell A , Cassidy K , Klingeman D , Yang Z , Stahler E , Smith S , Stahler D , Podar M . From wolves to humans: oral microbiome resistance to transfer across mammalian hosts . mBio. 2024;15(3):e0334223. 10.1128/mbio.03342-23 PMC10936156 38299854

[153] Razali K , Kaidi R , Abdelli A , et al. Oral flora of stray dogs and cats in Algeria: pasteurella and other zoonotic bacteria. Vet World. 2020;13(12):2806–2814. doi: 10.14202/vetworld.2020.2806-2814 PMC7811559 33488002

[154] Jamshidi S , Mozafari M , Machiani MK , Bashizade M . Comparative molecular detection of helicobacter spp. and wolinella in the oral cavity of cats and dogs with periodontal disease and healthy individuals . Iran J Microbiol. 2025;17(5):718–724. 10.18502/ijm.v17i5.19880 PMC12606375 41234637

[155] Portilho FVR , Nóbrega J , de Almeida BO , et al. Microbial complexity of oral cavity of healthy dogs identified by mass spectrometry and next-generation sequencing. Animals (Basel). 2023;13(15):2467. doi: 10.3390/ani13152467 PMC10417733 37570276

[156] Pu CY , Seshadri M , Manuballa S , et al. The oral microbiome and lung diseases. Curr Oral Health Rep. 2020;7(1):79–86. doi: 10.1007/s40496-020-00259-1

[157] Fellows Yates JA , Velsko IM , Aron F , Posth C , Hofman CA , Austin RM . The evolution and changing ecology of the African hominid oral microbiome. Proc Natl Acad Sci U S A. 2021;118(20):1–11. 10.1073/pnas.2021655118 PMC8157933 33972424

[158] Anju VT , Busi S , Imchen M , et al. Polymicrobial infections and biofilms: clinical significance and eradication strategies. Antibiotics (Basel). 2022;11(12):1731. doi: 10.3390/antibiotics11121731 PMC9774821 36551388

[159] Mahdizade Ari M , Mirkalantari S , Darban-Sarokhalil D , et al. Investigating the antimicrobial and anti-inflammatory effects of lactobacillus and bifidobacterium spp. On cariogenic and periodontitis pathogens. Front Microbiol. 2024;15:2024. doi: 10.3389/fmicb.2024.1383959 PMC11177620 38881669

[160] Rajasekaran JJ , Krishnamurthy HK , Bosco J , et al. Oral microbiome: a review of its impact on oral and systemic health. Microorganisms. 2024;12(9):1797. doi: 10.3390/microorganisms12091797 PMC11434369 39338471

[161] Xu HS , Roberts N , Singleton FL , et al. Survival and viability of nonculturableEscherichia coli andVibrio cholerae in the estuarine and marine environment. Microb Ecol. 1982;8(4):313–323. doi: 10.1007/BF02010671 24226049

[162] McDougald D , Rice SA , Weichart D , et al. Nonculturability: adaptation or debilitation? FEMS Microbiol Ecol. 1998;25(1):1–9. doi: 10.1111/j.1574-6941.1998.tb00455.x

[163] Sedgley CM , Lennan SL , Appelbe OK . Survival of enterococcus faecalis in root canals ex vivo. Int Endod J. 2005;38(10):735–742. doi: 10.1111/j.1365-2591.2005.01009.x 16164688

[164] Pasquaroli S , Zandri G , Vignaroli C , et al. Antibiotic pressure can induce the viable but non-culturable state in staphylococcus aureus growing in biofilms. J Antimicrob Chemother. 2013;68(8):1812–1817. doi: 10.1093/jac/dkt086 23515246

[165] Liu F , Ma R , Wang Y , et al. The clinical importance of campylobacter concisus and other human hosted campylobacter species. Front Cell Infect Microbiol. 2018;8:2018. doi: 10.3389/fcimb.2018.00243 PMC6066527 30087857

[166] Hirukawa S , Sagara H , Kaneto S , et al. Characterization of morphological conversion of helicobacter pylori under anaerobic conditions. Microbiol Immunol. 2018;62(4):221–228. doi: 10.1111/1348-0421.12582 29446491

[167] Nilsson HO , Blom J , Al-Soud WA , et al. Effect of cold starvation, acid stress, and nutrients on metabolic activity of helicobacter pylori. Appl Environ Microbiol. 2002;68(1):11–19. doi: 10.1128/AEM.68.1.11-19.2002 PMC126563 11772603

[168] Jee MJ , Tae CH , Han K , et al. Correlative light and electron microscopy visualization of helicobacter pylori in human saliva. Microsc Microanal. 2026;32(4):455–464. doi: 10.1093/mam/ozag061 42397351

[169] Percival SL , Suleman L , Vuotto C , et al. Healthcare-associated infections, medical devices and biofilms: risk, tolerance and control. J Med Microbiol. 2015;64(4):323–334. doi: 10.1099/jmm.0.000032 25670813

[170] Sharma S , Mohler J , Mahajan SD , et al. Microbial biofilm: a review on formation, infection, antibiotic resistance, control measures, and innovative treatment. Microorganisms. 2023;11(6):1614. doi: 10.3390/microorganisms11061614 PMC10305407 37375116

[171] Thurnheer T , Belibasakis GN . Integration of non-oral bacteria into in vitro oral biofilms. Virulence. 2015;6(3):258–264. doi: 10.4161/21505594.2014.967608 PMC4601515 25483866

[172] Vieira Colombo AP , Magalhães CB , Hartenbach FARR , et al. Periodontal-disease-associated biofilm: a reservoir for pathogens of medical importance. Microb Pathog. 2016;94:27–34. doi: 10.1016/j.micpath.2015.09.009 26416306

[173] Bassotti G , Fruganti A , Stracci F , et al. Cytotoxic synergism of clostridioides difficile toxin B with proinflammatory cytokines in subjects with inflammatory bowel diseases. World J Gastroenterol. 2023;29(4):582–596. doi: 10.3748/wjg.v29.i4.582 PMC9896618 36742168

[174] Frost LR , Cheng JKJ , Unnikrishnan M . Clostridioides difficile biofilms: a mechanism of persistence in the gut? PLoS Pathog. 2021;17(3):e1009348. doi: 10.1371/journal.ppat.1009348 PMC7951904 33705497

[175] Espíndola LCP , do Nascimento MVMR , do Souto RM , et al. Antimicrobial susceptibility and virulence of enterococcus spp. Isolated from periodontitis-associated subgingival biofilm. J Periodontol. 2021;92(11):1588–1600. doi: 10.1002/JPER.20-0815 33650677

[176] van Leeuwen PT , van der Peet JM , Bikker FJ , et al. Interspecies interactions between clostridium difficile and candida albicans. mSphere. 2016;1(6):1–15. doi: 10.1128/mSphere.00187-16 PMC5103046 27840850

[177] Valadbeigi H , Khoshnood S , Negahdari B , et al. Mixed oral biofilms are controlled by the interspecies interactions of fusobacterium nucleatum. Oral Dis. 2024;30(6):3582–3590. doi: 10.1111/odi.14822 38009960

[178] Sakanaka A , Kuboniwa M , Shimma S , Alghamdi CA , Mayumi S , Lamont RJ , Fukusaki E , Amano A . Fusobacterium nucleatum metabolically integrates commensals and pathogens in oral biofilms . mSystems. 2022;7(4):e0017022. 10.1128/msystems.00170-22 PMC9426547 35852319

[179] He X , Hu W , Kaplan CW , et al. Adherence to streptococci facilitates fusobacterium nucleatum integration into an oral microbial community. Microb Ecol. 2012;63(3):532–542. doi: 10.1007/s00248-011-9989-2 PMC3313671 22202886

[180] Gao Y , Ma L , Su J . Host and microbial-derived metabolites for clostridioides difficile infection: contributions, mechanisms and potential applications. Microbiol Res. 2022;263:127113. doi: 10.1016/j.micres.2022.127113 35841835

[181] Semenyuk EG , Laning ML , Foley J , et al. Spore formation and toxin production in clostridium difficile biofilms. PLoS One. 2014;9(1):e87757. doi: 10.1371/journal.pone.0087757 PMC3907560 24498186

[182] Hall MW , Singh N , Ng KF , et al. Inter-personal diversity and temporal dynamics of dental, tongue, and salivary microbiota in the healthy oral cavity. NPJ Biofilms Microbiomes. 2017;3(1):2. doi: 10.1038/s41522-016-0011-0 PMC5445578 28649403

[183] Baquero F , Coque TM , Galán JC , et al. The origin of niches and species in the bacterial world. Front Microbiol. 2021;12:657986. doi: 10.3389/fmicb.2021.657986 PMC8010147 33815348

[184] Proctor DM , Relman DA . The landscape ecology and microbiota of the human nose, mouth, and throat. Cell Host Microbe. 2017;21(4):421–432. doi: 10.1016/j.chom.2017.03.011 PMC5538306 28407480

[185] Mark Welch JL , Ramírez-Puebla ST , Borisy GG . Oral microbiome geography: micron-scale habitat and niche. Cell Host Microbe. 2020;28(2):160–168. doi: 10.1016/j.chom.2020.07.009 PMC7604680 32791109

[186] Dos Santos PGST , Silva LB , Valença PAM , Cunha RSC , Ataíde Filho AC , Silveira FMM , Fonseca CSM . Mechanisms and substances involved in inflammation in the oral cavity–Literature review. Res Soc Dev. 2024;13(7):e11913746429.

[187] Kolenbrander PE , Palmer RJ , Periasamy S , et al. Oral multispecies biofilm development and the key role of cell-cell distance. Nat Rev Microbiol. 2010;8(7):471–480. doi: 10.1038/nrmicro2381 20514044

[188] Iroha IR , Mohammed ID , Moses IB , et al. Molecular characterization of enterobacteriacea isolated from gingivitis and periodontitis patients and the antimicrobial activity of mouth wash agents. Scientific African. 2022;15:e01106. doi: 10.1016/j.sciaf.2022.e01106

[189] Hajishengallis G . The inflammophilic character of the periodontitis-associated microbiota. Mol Oral Microbiol. 2014;29(6):248–257. doi: 10.1111/omi.12065 PMC4232466 24976068

[190] Marsh PD . Are dental diseases examples of ecological catastrophes? Microbiology. 2003;149(Pt 2):279–294. doi: 10.1099/mic.0.26082-0 12624191

[191] Sousa V , Nibali L , Spratt D , et al. Peri-implant and periodontal microbiome diversity in aggressive periodontitis patients: a pilot study. Clin Oral Implants Res. 2017;28(5):558–570. doi: 10.1111/clr.12834 27170047

[192] Faisal RH , Ali AO . Increased bacterial load of filifactor alocis in deep periodontal pockets discriminate between periodontitis stage 3 and 4. Front Oral Health. 2025;6:1543030. doi: 10.3389/froh.2025.1543030 PMC11983625 40213514

[193] Al-Ahmad A , Muzafferiy F , Anderson AC , et al. Shift of microbial composition of peri-implantitis-associated oral biofilm as revealed by 16S rRNA gene cloning. J Med Microbiol. 2018;67(3):332–340. doi: 10.1099/jmm.0.000682 29458668

[194] Charalampakis G , Belibasakis GN . Microbiome of peri-implant infections: lessons from conventional, molecular and metagenomic analyses. Virulence. 2015;6(3):183–187. doi: 10.4161/21505594.2014.980661 PMC4601299 25654499

[195] Shevela TL , Pohodenko-Chudakova IO , Koctiuk SA . Algorithm for the diagnosis of helicobacter pylori with peri-implantitis. СТОМАТОЛОГ. 2017;26:28–34.

[196] Kumar PS , Mason MR , Brooker MR , et al. Pyrosequencing reveals unique microbial signatures associated with healthy and failing dental implants. J Clin Periodontol. 2012;39(5):425–433. doi: 10.1111/j.1600-051X.2012.01856.x PMC3323747 22417294

[197] Cheng X , He F , Si M , et al. Effects of antibiotic use on saliva antibody content and oral microbiota in sprague dawley rats. Front Cell Infect Microbiol. 2022;12:2022. doi: 10.3389/fcimb.2022.721691 PMC8843035 35174102

[198] Buswell CM , Herlihy Y , Marsh P , et al. Coaggregation amongst aquatic biofilm bacteria. J Appl Microbiol. 1997;83(4):477–484. doi: 10.1046/j.1365-2672.1997.00260.x

[199] Vander Haar EL , So J , Gyamfi-Bannerman C , et al. Fusobacterium nucleatum and adverse pregnancy outcomes: epidemiological and mechanistic evidence. Anaerobe. 2018;50:55–59. doi: 10.1016/j.anaerobe.2018.01.008 PMC6750227 29409815

[200] Kövér Z , Gajdács M , Polgár B , et al. The microbiological background of medication-related osteonecrosis of the jaw (MRONJ): clinical evidence based on traditional culture and molecular biological detection methods. Antibiotics (Basel). 2025;14(2):203. doi: 10.3390/antibiotics14020203 PMC11851722 40001446

[201] Hinson AM , Siegel ER , Stack BC . Is bisphosphonate-related osteonecrosis of the jaw an infection? A histological and microbiological ten-year summary. Int J Dent. 2014;2014:452737. 10.1155/2014/452737 PMC4095654 25089126

[202] Aftimos V , Zeinoun T , Bou Tayeh R , Aftimos G . Bisphosphonate related osteonecrosis of the jaw: a study of 18 cases associated with fungal infection. Int J Dent. 2014;2014:869067. 10.1155/2014/869067 PMC3948474 24696686

[203] Souza LCD , Lopes FF , Bastos EG , et al. Oral infection by pseudomonas aeruginosa in patient with chronic kidney disease - a case report. J Bras Nefrol. 2018;40(1):82–85. doi: 10.1590/1678-4685-jbn-3812 PMC6533965 29796582

[204] Sedghizadeh PP , Yooseph S , Fadrosh DW , et al. Metagenomic investigation of microbes and viruses in patients with jaw osteonecrosis associated with bisphosphonate therapy. Oral Surg Oral Med Oral Pathol Oral Radiol. 2012;114(6):764–770. doi: 10.1016/j.oooo.2012.08.444 PMC3556897 23159114

[205] Ganguli A , Steward C , Butler SL , et al. Bacterial adhesion to bisphosphonate coated hydroxyapatite. J Mater Sci Mater Med. 2005;16(4):283–287. doi: 10.1007/s10856-005-0625-x 15803271

[206] Papathanakos G , Andrianopoulos I , Xenikakis M , et al. Clinical sepsis phenotypes in critically ill patients. Microorganisms. 2023;11(9):2165. doi: 10.3390/microorganisms11092165 PMC10538192 37764009

[207] Hou F , Qiao Y , Shi Y , et al. A retrospective analysis comparing metagenomic next-generation sequencing with conventional microbiology testing for the identification of pathogens in patients with severe infections. Front Cell Infect Microbiol. 2025;15:2025. doi: 10.3389/fcimb.2025.1530486 PMC12011730 40264936

[208] Siqueira Jr. JF , Rôças IN , Alves FR , et al. Bacteria in the apical root canal of teeth with primary apical periodontitis. Oral Surg Oral Med Oral Pathol Oral Radiol Endod. 2009;107(5):721–726. doi: 10.1016/j.tripleo.2009.01.042 19426923

[209] Samson CA , Whitford W , Snell RG , et al. Contaminating DNA in human saliva alters the detection of variants from whole genome sequencing. Sci Rep. 2020;10(1):19255. doi: 10.1038/s41598-020-76022-4 PMC7648094 33159102

[210] Ghapanchi J , Bazargani A , Khorshidi H , Erfani M , Erfani F , Azad A , Derafshi R , Kalantari AH . Isolation and identification of Non- commensal pathogenic bacteria in the saliva of patients candidate for liver transplant: a cross sectional study in shiraz, south of Iran. J Dent (Shiraz). 2020;21(2):81–86. 10.30476/DENTJODS.2019.77854. PMC7280545 32582821

[211] Tsuzukibashi O , Uchibori S , Kobayashi T , et al. A selective medium for the isolation of microbacterium species in oral cavities. J Microbiol Methods. 2015;116:60–65. doi: 10.1016/j.mimet.2015.06.016 26149625

[212] Zhang M , Zhao Y , Umar A , et al. Comparative analysis of microbial composition and functional characteristics in dental plaque and saliva of oral cancer patients. BMC Oral Health. 2024;24(1):411. doi: 10.1186/s12903-024-04181-1 PMC10993480 38575895

[213] Derafshi R , Bazargani A , Ghapanchi J , et al. Isolation and identification of nonoral pathogenic bacteria in the oral cavity of patients with removable dentures. J Int Soc Prev Community Dent. 2017;7(4):197–201. doi: 10.4103/jispcd.JISPCD_90_17 PMC5558254 28852636

[214] Rautemaa-Richardson R , A Van der Reijden Wa W , Dahlen G , et al. Quality control for diagnostic oral microbiology laboratories in european countries. J Oral Microbiol. 2011;3:8395. doi: 10.3402/jom.v3i0.8395 PMC3213926 22084647

[215] Sukumar S , Roberts A , Martin F , et al. Metagenomic insights into transferable antibiotic resistance in oral bacteria. J Dent Res. 2016;95(9):969–976. doi: 10.1177/0022034516648944 27183895

[216] Cieplik F , Scholz KJ , Al-Ahmad A . Antimicrobial resistance–a critical and timely challenge for dentistry and oral healthcare: Antimicrobial resistance–a critical and timely challenge for dentistry and oral healthcare. UK London: Nature Publishing Group; 2025. 10.1038/s41415-025-9380-1 41315698

[217] Neuta Y , Leguizamon N , Pajaro P , et al. Molecular and phenotypic evaluation of antibiotic resistance in enteric rods isolated from the oral cavity. Antibiotics (Basel). 2025;14(6):564. doi: 10.3390/antibiotics14060564 PMC12189819 40558154

[218] Sukumar S , Wang F , Simpson CA , et al. Development of the oral resistome during the first decade of life. Nat Commun. 2023;14(1):1291. doi: 10.1038/s41467-023-36781-w PMC9998430 36894532

[219] Brooks L , Narvekar U , McDonald A , et al. Prevalence of antibiotic resistance genes in the oral cavity and mobile genetic elements that disseminate antimicrobial resistance: a systematic review. Mol Oral Microbiol. 2022;37(4):133–153. doi: 10.1111/omi.12375 35674142

[220] Michaelis C , Grohmann E . Horizontal gene transfer of antibiotic resistance genes in biofilms. Antibiotics (Basel). 2023;12(2):328. doi: 10.3390/antibiotics12020328 PMC9952180 36830238

[221] Roberts AP , Pratten J , Wilson M , et al. Transfer of a conjugative transposon, Tn5397 in a model oral biofilm. FEMS Microbiol Lett. 1999;177(1):63–66. doi: 10.1111/j.1574-6968.1999.tb13714.x 10436923

[222] Vickerman MM , Mansfield JM . Streptococcal peptides that signal enterococcus faecalis cells carrying the pheromone-responsive conjugative plasmid pAM373. Mol Oral Microbiol. 2019;34(6):254–262. doi: 10.1111/omi.12271 31610092

[223] Santibañez-Bedolla KE , Orozco-Uriarte MJ , Alvarez-Canales JA , et al. Oral colonization by gram-negative bacilli in patients with hematologic malignancies and solid tumors compared with healthy controls. BMC Oral Health. 2023;23(1):465. doi: 10.1186/s12903-023-03172-y PMC10329337 37422668

[224] Katkowska M , Garbacz K , Kwapisz E , et al. High oral carriage of multidrug resistant gram-negative bacilli in adolescents: the SOPKARD-Junior study. Front Cell Infect Microbiol. 2023;13:2023. doi: 10.3389/fcimb.2023.1265777 PMC10687414 38035342

[225] Almeida VSM , Azevedo J , Leal HF , et al. Bacterial diversity and prevalence of antibiotic resistance genes in the oral microbiome. PLoS One. 2020;15(9):e0239664. doi: 10.1371/journal.pone.0239664 PMC7523989 32991620

